# Exploring the Role of an Electrolyte Additive in Suppressing Surface Reconstruction of a Ni-Rich NMC Cathode at Ultrahigh Voltage via Enhanced In Situ and Operando Characterization Methods

**DOI:** 10.1021/acsami.3c15670

**Published:** 2024-02-09

**Authors:** Huidong Dai, Luisa Gomes, Derrick Maxwell, Somayeh Zamani, Kevin Yang, Dianne Atienza, Nilesh Dale, Sanjeev Mukerjee

**Affiliations:** † Department of Chemistry and Chemical Biology, Northeastern University, 360 Huntington Avenue, Boston, Massachusetts 02115, United States; ‡ Department of Chemical Engineering, Northeastern University, 360 Huntington Avenue, Boston, Massachusetts 02115, United States; § Nissan Technical Center North America, 39001 Sunrise Drive, Farmington Hills, Michigan 48331, United States

**Keywords:** CEI layer, rock-salt formation, NMC cathodes, in situ/operando Raman, in situ XAS

## Abstract

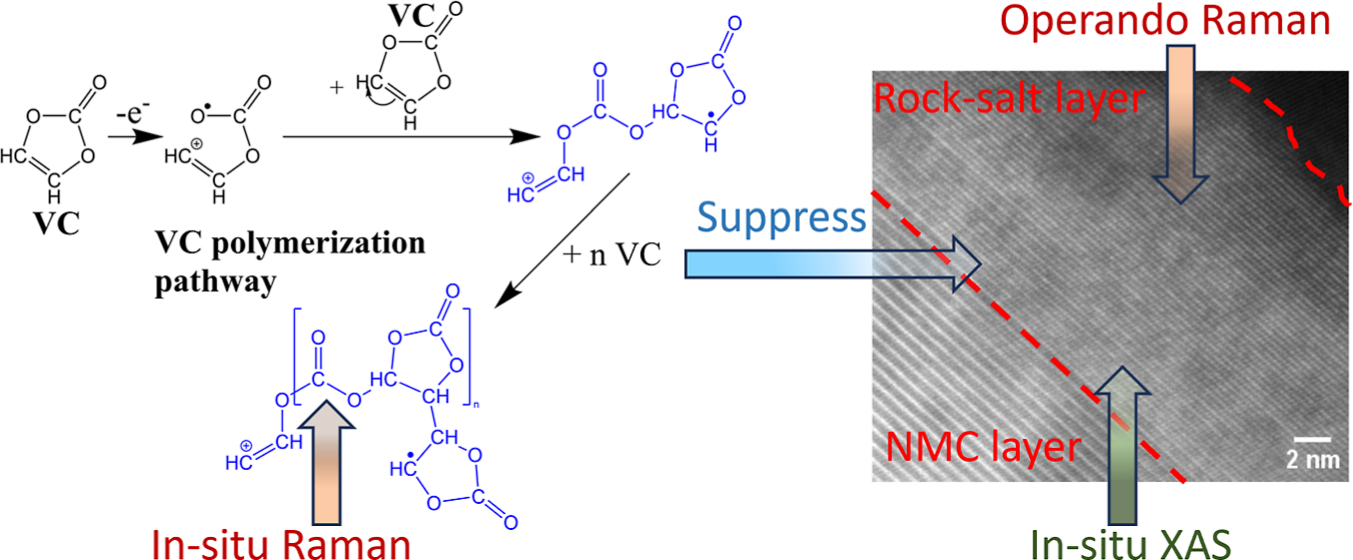

Vinylene carbonate (VC) is a widely used electrolyte additive in lithium-ion batteries for enhanced solid electrolyte interphase formation on the anode side. However, the cathode electrolyte interphase (CEI) formation with VC has received a lot less attention. This study presents a comprehensive investigation employing advanced in situ/operando-based Raman and X-ray absorption spectroscopy (XAS) to explore the effect of electrolyte composition on the CEI formation and suppression of surface reconstruction of Li_*x*_Ni_*y*_Mn_*z*_Co_1–*y*–*z*_O_2_ (NMC) cathodes. A novel chemical pathway via VC polymerization is proposed based on experimental results. In situ Raman spectra revealed a new peak at 995 cm^–1^, indicating the presence of C–O semi-carbonates resulting from the radical polymerization of VC. Operando Raman analysis unveiled the formation of NiO at 490 cm^–1^ in the baseline system under ultrahigh voltage (up to 5.2 V). However, this peak was conspicuously absent in the VC electrolyte, signifying the effectiveness of VC in suppressing surface reconstruction. Further investigation was carried out utilizing in situ XAS compared X-ray absorption near edge structure spectra from cells of 3 and 20 cycles in both electrolytes at different operating voltages. The observed shift at the Ni K-edge confirmed a more substantial reduction of Ni in the baseline electrolyte compared to that in the VC electrolyte, thus indicating less CEI protection in the former. A sophisticated extended X-ray absorption fine structure analysis quantitatively confirmed the effective suppression of rock-salt formation with the VC electrolyte during the charging process, consistent with the operando Raman results. The in situ XAS results thus provided additional support for the key findings of this study, establishing the crucial role of VC polymerization in enhancing CEI stability and mitigating surface reconstruction on NMC cathodes. This work clarifies the relationship between the enhanced CEI layer and NMC degradation and inspires rational electrolyte design for long-cycling NMC cathodes.

## 1 Introduction

Lithium-ion batteries (LIBs) have emerged as a prominent clean energy storage technology that plays a pivotal role in the proliferation of renewable energy systems. In the face of competing technologies such as hydrogen energy, wind energy, and solar energy, LIBs have proved to be a leading contender in the quest for sustainable energy storage solutions.^1−5^ Among the various cathode active materials, Li_*x*_Ni_*y*_Mn_*z*_Co_1–*y*–*z*_O_2_ (NMC) has garnered great attention due to its high capacity^1,6^ and superior operation voltage window.^1^ However, NMC also faces some challenges when operating at high voltages and temperatures such as oxygen release from the NMC structure, which can cause structural degradation, capacity fading, and safety issues.^7^ Among various NMC cathode compositions, LiNi_0.8_Mn_0.1_Co_0.1_O_2_ (NMC811) stands out as the most attractive option due to its lower manganese and cobalt content than other NMC phases (NMC111 or NMC622). However, despite its advantages, NMC811 encounters significant capacity degradation during cycling, particularly when operated at high voltages of up to 4.8 V (vs Li/Li^+^). This deterioration significantly hinders its practical application and can be primarily attributed to the formation of spinel and/or rock-salt phases on the cathode particle surface during the cycling process.^8^ The degradation of the cathode is mainly associated with bulk structural instability and surface electrochemical deterioration of NMC particles. The subsequent migration of transition metal ions (TMs) to the neighboring Li slabs triggers the irreversible phase transition of the overall layered NMC framework to spinel and/or rock-salt structures and particle cracking induced by the stress from Li^+^ extraction and insertion.^9−12^ Although several strategies have been proposed to improve the cycling performance of NMC cathodes, including coating with protective layers such as Li_2_ZrO_3_,^13,14^ optimizing core–shell structure and element concentration gradient,^15^ or optimizing the charge cutoff voltage,^16^ the spinel and/or rock-salt formation remains one of the most severe problems, leading to poor cycle stability and fast capacity decay. Therefore, a detailed understanding of the rock-salt formation mechanism and the factors affecting it is essential to enhance the performance and stability of NMC cathodes.

The electrode–electrolyte interface (EEI) is important to study because the solid–electrolyte interface (SEI) is formed there via electrochemical and chemical processes that result from electrolyte decomposition when exposed to oxidative potentials. The cathode–electrolyte interphases (CEI), however, have received less attention, even though it is widely believed that a uniform and cohesive CEI layer can provide cathode protection by inhibiting transition metal mixing and surface reconstruction even at high operating voltage window up to 4.8 V (vs Li/Li^+^).^1,17,18^ While several cathodes operate within the thermodynamic stability window of commercial carbonate electrolytes, the rich surface chemistry of alkyl carbonate electrolytes, acid–base reactions, nucleophilic reactions, induced polymerizations, and transition metal dissolution make the CEI formation process complex.^19−21^ After formation, the CEI can block contact between the electrolyte and active materials, thus reducing ongoing side reactions or corrosion and minimizing capacity degradation and safety issues. A stable CEI layer should be ionically conducting, have optimal thickness, and facilitate efficient Li^+^ ion transport to ensure superior electrochemical performance.^22^ The current CEI layer in most Li-NMC batteries is formed through the decomposition of carbonate solvents, such as ethylene carbonate (EC) or diethyl carbonate (DEC) due to their high operation voltage window and dielectric constants.^23^ However, this choice of solvent also leads to the formation of a porous and inhomogeneous CEI layer. This leaves TMs susceptible to attack by hydrofluoric acid (from the decomposition of LiPF_6_ that occurs in parallel). Therefore, the formation of rock-salt structures on the surface of the NMC is exacerbated.

Extensive explorations of electrolyte additives in academia and industry have been pursued to bolster SEI and CEI compositions for improved cell-level performance. These additives bring value to the electrolyte package, because they provide additional resilience by forming stable interfaces with low relative percentages (sometimes less than 1% of total electrolyte). Additives such as fluoroethylene carbonate,^24^ ethylene sulfite,^25^ and isocyanates^26^ have been extensively investigated for this purpose. Among the various additives, vinylene carbonate (VC) has garnered significant attention and is commonly employed to enhance the efficacy of CEI formation.^27−32^ VC undergoes oxidation at the cathode electrode at a lower potential than other carbonate solvents, resulting in the generation of insoluble products that contribute to the formation of an effective CEI.^19,33^ The oxidation products of VC contain carbon double bonds that can further undergo polymerization, resulting in the formation of a polymeric surface species.^27,34^ The surface films present several promising advantages, including greater cohesion and flexibility, improving the surface passivation.^29,30^ However, the mechanism of CEI formation through VC oxidation and polymerization is not widely understood. Additionally, there is a lack of compelling evidence regarding the stabilizing effect on cycled NMC cathodes from VC-induced CEI modifications at high voltages (4.8 V and higher), where rock-salt formation is more likely to occur.

State-of-the-art techniques have been previously employed in the literature to investigate the thin layer of CEI and rock-salt reconstruction on the cathode: X-ray photoelectron spectroscopy (XPS),^1,19,30,35^ scanning electron microscope (SEM) coupled with energy-dispersive X-ray spectroscopy,^10,35,36^ transmission electron microscopy (TEM) or scanning transmission electron microscopy (STEM),^1,10,19,37^ and time-of-flight secondary ion mass spectrometry.^38,39^ Li et al. utilized in situ surface enhanced Raman spectroscopy to observe the surface chemistry evolution on a baseline electrolyte NMC cathode.^40^ This technique provides a high sensitivity for identifying near-surface chemistry. However, it requires doping noble metal nanoparticles, such as Au, onto the sample surface, which is limited by the substrate material and the morphology of the surface. Therefore, the enhanced Raman signals have been called into question by some members of the scientific community due to the required usage of roughened metals.^40,41^ While these techniques offer valuable insights into the composition and formation pathways of the CEI, their application is predominantly confined to ex situ conditions, which restricts their ability to examine the on-site redox reactions occurring between the electrolyte and electrode. Additionally, only a limited number of research groups have utilized in situ/operando approaches to unveil the CEI layer’s impact on surface stabilization and rock-salt formation prevention. Arguments have also observed concerning possible artifacts introduced in the ex situ measurements due to electrode harvesting, sample preparation, and side reactions when exposed to the environment.^42^

This study seeks to obtain a more comprehensive and accurate understanding of the CEI formation and its corresponding NMC surface stabilization effect with modified CEI protection. A series of in situ/operando-based Raman spectroscopy and X-ray absorption spectroscopy (XAS) characterizations were employed. XPS studies have effectively demonstrated that LiCO_3_ is one of the major components in the CEI of a Li-NMC battery, accompanied by trace compounds such as LiF, LiPF_*x*_, and LiPO_*x*_F_*y*_.^19,30^ Additional fluorinated additives like lithium difluorophosphate or tris(2,2,2-trifluoroethyl) orthoformate (TFEO) also introduce Li_3_PO_4_ as a significant composition in the CEI layer, as confirmed by XPS.^1,35^ These chemical compounds form during the decomposition of solvents and additives throughout cycling, interacting with LiPF_6_ decomposition, particularly under extreme conditions like ultrahigh operation voltage or prolonged cycle depth. Despite VC’s extensive history as an electrolyte additive, gaining a comprehensive understanding of its role in CEI formation is an area that requires further exploration. This is especially true with regard to its consequential impact on NMC surface stabilization and the underlying mechanisms. The surface sensitivity of Raman spectroscopy and the ability of XAS to detect local atom structural changes make them well-suited for interface characterization. This pairing is particularly beneficial for exploring the role of VC additives in LiBs. By conducting a thorough comparison of the surface chemistry between the baseline and VC containing electrolytes, this study showcased VC as an excellent candidate for NMC surface passivation. Specifically, VC exhibits an earlier oxidation response (around 4.2 V) in a half cell than other carbonate solvents during the initial cycles, resulting in the formation of a cohesive and less-porous polymeric layer on the NMC cathode surface. Consequently, the modified CEI layer formed through VC polymerization plays a crucial role in effectively suppressing the transformation of NMC from layered to spinel and/or rock-salt structures, even under ultrahigh operation voltages (up to 5.2 V). The results of this study provide valuable insights into the characterization of advanced electrolyte systems and complex chemistry environments in LIBs cathodes, which can guide the design and development of new LIBs systems.

## 2 Methods

### 2.1 NMC Electrode Preparation

The composite NMC811 (from MSE Supplies LLC) electrodes were fabricated by dispersing the cathode active materials (92 wt %), poly(vinylidene fluoride) (PVDF) (5 wt %, from Fisher Scientific), and carbon black (3 wt %, Ketjenblack from MSE Supplies LLC) in *N*-methyl-2-pyrrolidone (NMP) (from Sigma-Aldrich). The resulting slurry mixture was prepared with a solid ratio of 55–60 wt % and mixed in a dual asymmetric centrifugal mixer (DAC 330-100 Pro, from FlackTek Inc.). The slurry was subsequently applied onto a 25 μm thick separator (Celgard 2325) for in situ/operando Raman spectroscopy characterization. For the coin cell (CR2032) assembly, the slurry was coated onto a 16 μm thick aluminum foil (from MTI Corp.) with a cathode loading around 12 mg/cm^2^. The cathode thickness was kept at 120 μm for both in situ/operando Raman spectroscopy and coin cell assembly.

The cathode materials used in the in situ XAS cells initially underwent a sieving procedure through a 5 μm mesh size sieve (Micro-Mesh, from Industrial Netting) to obtain ultrafine powders. Subsequently, the same mixing procedure was applied, and the slurry was coated onto 16 μm thick aluminum foil (from MTI Corp.). The coating thickness was carefully controlled at 5 to 7 μm to ensure the formation of a thin electrode layer suitable for XAS analysis.

The electrodes on the separator were punched into 10 mm circles for in situ/operando Raman cell assembly, and electrodes on the aluminum were punched into 12.7 mm circles for in situ XAS cell assembly. The as-punched electrodes for the in situ/operando Raman cell and in situ XAS cell were dried at 90 and 120 °C overnight, respectively, in a fume hood and then immediately transferred to the glovebox to maintain a controlled atmosphere.

### 2.2 In Situ/Operando Raman Spectroscopy and In Situ XAS

The Raman spectroscopy experiments were performed using a confocal Raman microscope (XploRA Plus, from Horiba Scientific) equipped with a 50× objective lens and configured with multiple wavelength lasers ranging from 473 to 732 nm. The spectroscopy cell (ECC-Opto-10, from El-Cell) employed for Raman analysis featured a 0.2 mm thick sapphire as an optical window, which is depicted in Figure S1a in the Supporting Information, with a schematic illustration presented in Figure S1b. For CEI characterization on the cathode surface, a 732 nm laser with a power of 0.3 mW was carefully selected to ensure that the laser frequency was tuned to a more surface-sensitive configuration. This choice of laser power was also sufficient to deliver the required intensity through the optical window. Subsequently, a 473 nm laser with a power of 2.5 mW was employed for the characterization of rock-salt formation on the NMC surface.

The XAS cell was fashioned using two 40 mm diameter aluminum plates (grade 6061) as the housing structure to exert adequate pressure, while a 3 × 12 mm slit with a Kapton window on each side was utilized to allow the passage of the synchrotron beam. To accommodate the electrode stack, five layers of a 70 μm thick gasket (1007 silicone-coated fiberglass, from Saint-Gobain) with a central hole of 19.05 mm diameter were utilized. The positioning of the aluminum plates and gaskets was secured using eight sets of 12 mm long screws and nuts with 8/32 threads. Additionally, a thermal shrink plastic was used as insulation to cover the portion where the screw contacted the aluminum plates. For visual reference, Figure S1c–e shows three views of the XAS cell, and a schematic illustration is presented in Figure S1f. Two sets of XAS cells were meticulously prepared for this investigation. The first set, referred to as “formation” cells, was assembled on-site at the XAS facility and underwent three subsequent cycles of *C*/10 (*C* represents the full capacity of the LIBs discharged in one h) between 2.8 and 4.3 V (vs Li/Li^+^) prior to XAS measurements to ensure a well-developed CEI on the cathode. The second set, known as “cycled” cells, was assembled in our laboratory and had undergone 20 cycles under similar conditions before XAS measurements. The cells were cycled between 2.8 to 4.8 V to simulate the ultrahigh voltage scenario during the in situ XAS experiments. The XAS spectra of the nickel (Ni), manganese (Mn), and cobalt (Co) K-edge were captured under in situ conditions at their open circuit potential (OCP) and when charged to 4.3 and 4.8 V, respectively. The XAS experiments were carried out at beamline station 7-BM quick X-ray absorption and scattering located at the National Synchrotron Light Source II (NSLS-II) of Brookhaven National Laboratory.

The baseline electrolyte was 1 M LiPF_6_ in EC/DEC (50:50 volume) (from Fisher Scientific). Moreover, 0.5 and 2 wt % VC (from Fisher Scientific) were mixed with baseline electrolyte forming the additive electrolytes. To ensure a thorough in situ characterization of CEI formation, the Raman cells were filled with an ample amount of 100 μL of electrolyte, ensuring complete immersion of the cathode bulk region. For the rock-salt formation experiment in the Raman cell, a minimum volume of 20 μL was used to minimize any interference from the electrolyte. Regarding the XAS experiments, a volume of 50 μL was employed to reduce electrolyte interference. The Raman and XAS cells underwent a 12 h resting period prior to charging/discharging at a *C*/10 to minimize the overpotential penalty. All voltages reported in this study were referenced with respect to the Li counter electrode. Finally, to eliminate the interference of fluorescence, a fourth-order polynomial function was employed to background-correct the Raman spectra and a fifth-order polynomial smoothing method was utilized to enhance the signal-to-noise ratio while retaining the spectroscopic features using the LabSpec 6 Spectroscopy Suite software by Horiba Scientific. All XAS data were processed via the program ATHENA and ARTEMIS.^43^

### 2.3 Li-NMC Half Cell Assembly and Testing Procedure

The Li-NMC half cells were fabricated within coin cells made of stainless steel 316 (CR2032, from Gelon Lib Co., Ltd.). A combination of three layers of 25 μm thick Celgard (Celgard 2325) and an extra layer of 67 μm thick fiberglass (from Fisher Scientific) were used as the separator. Pure lithium discs with a thickness of 600 μm (from MTI Corp.) were used as anodes. A hydraulic press (TMAX-JKKF20-TC, from Tmax Battery Equipment Limited) was used for the coin cell assembly. The entire assembly procedure was conducted in the glovebox (from MBraun Inc., H_2_O content below 1 ppm and O_2_ content below 1 ppm) with an argon environment. All coin cells underwent a 12 h resting procedure prior to electrochemical investigation. A 12-channel battery testing station (BT-2143, from Arbin Instruments) was used to charge/discharge the coin cells with different *C*-rates between 2.8 and 4.3 V. Cyclic voltammetry (CV) experiments were performed using a potentiostat/galvanostat (VoltaLab PGP201, from Hach). The CVs were performed in a voltage range of 2.8 to 4.3 V versus Li/Li^+^ at a scan rate of 0.1 mV/s. For each cell, three CV cycles were performed. Electrochemical impedance spectroscopy (EIS) measurements were carried out using a potentiostat/galvanostat (AutoLab PGSTAT302N, from Metrohm) equipped with an impedance module and controlled by using NOVA 2.1 software. EIS measurements were performed at the OCP with coin cells in a frequency range of 100,000 to 0.001 Hz after 100 cycles at *C*/10.

### 2.4 Particle Dispersion Treatment for SEM and TEM

The cathodes utilized for SEM and TEM characterization were extracted from CR2032 coin cell configurations after 100 cycles at *C*/10. Pristine cathodes were subjected to characterization in their as-coated state without being assembled in coin cells. To ensure the removal of any residual salts and solvents, the cathodes were initially washed with an ethyl methyl carbonate solution and subsequently dried in the glovebox. SEM images were captured in the cathodes’ as-cycled condition after drying in the glovebox for 12 h. Subsequently, the cathodes were dissolved in *N*,*N*-dimethylformamide (DMF) and subjected to sonication to eliminate PVDF and ensure the dispersion of NMC particles. The resulting samples were then transferred to a drying oven and left overnight at 85 °C to effectively remove the solvent. For high-angle annular dark-field scanning transmission electron microscopy (HAADF-STEM) and TEM measurements, isopropanol was employed to further disperse the NMC particles, which were subsequently deposited on a copper grid coated with a lacey carbon film. The condition we used for STEM and TEM is at a high tension of 300 kV. The TEM model we used is a Titan Themis 300 S/TEM with a probe corrector.

## 3 Results and Discussion

### 3.1 Electrochemical Characterization of NMC Half Cells

**Figure 1:**
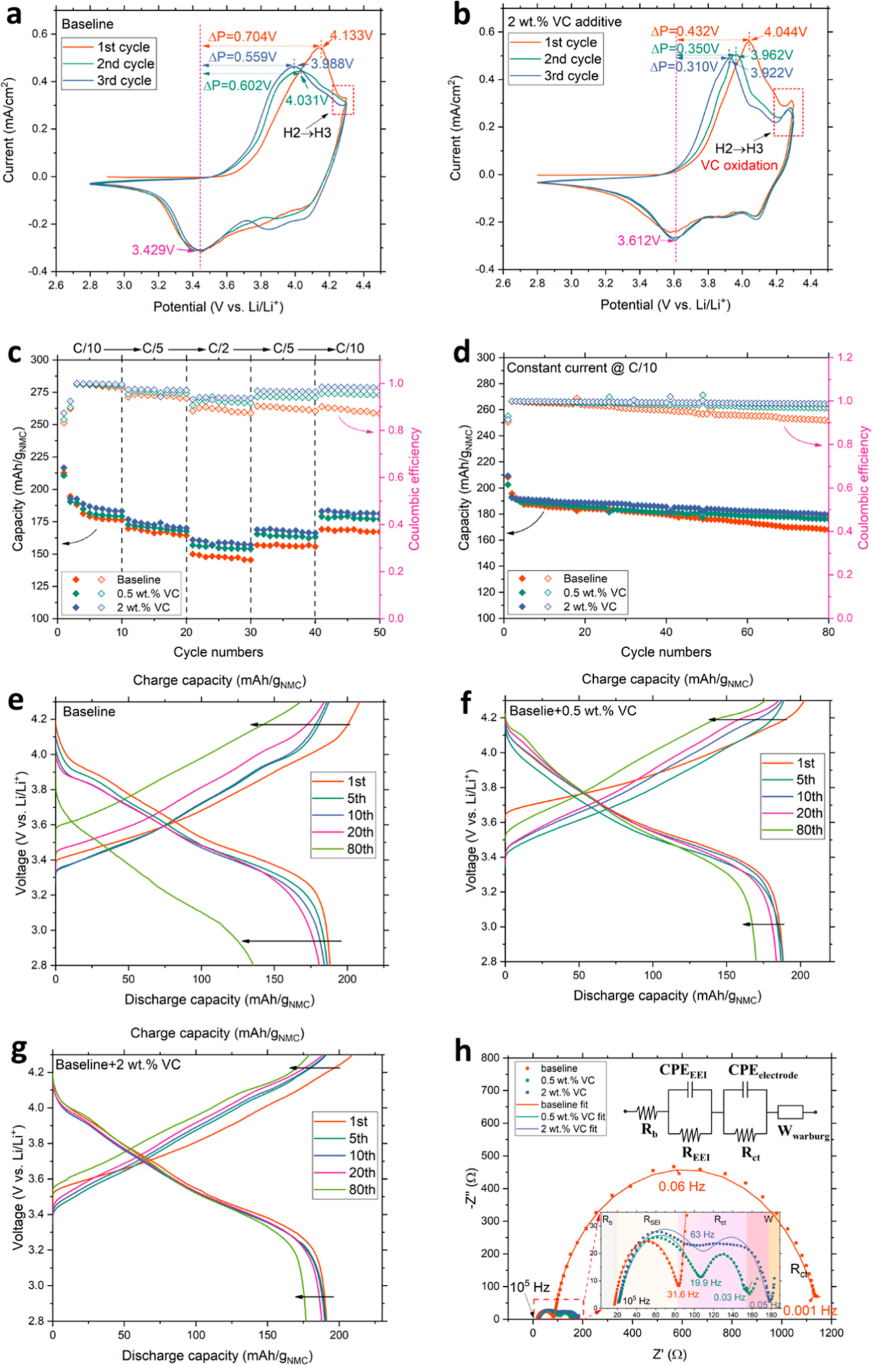
Preliminary electrochemical characterization results from coin cells. Panel (a) presents the CV results at a scan rate of 0.1 mV/s for the baseline electrolyte during the first 3 cycles, while panel (b) displays the CV results for the 2 wt % VC containing electrolyte under similar conditions. Panel (c) showcases the rate capability data for the baseline, 0.5 and 2 wt % VC containing electrolytes over 50 cycles. In panel (d), the cycling performance at constant current at *C*/10 was displayed over 80 cycles. Panels (e–g) represent the specific charge/discharge curves of the 1st, 5th, 10th, 20th, and 80th cycles from panel (d) for the baseline, 0.5 wt % VC, and 2 wt % VC containing electrolytes. In panel (h), EIS results are presented in scattered dots for the baseline, 0.5 wt %, and 2 wt % VC containing electrolytes after 100 constant-current cycles. The ECM is depicted in the upper right corner, and the solid lines represent the fitting results. The EIS measurements were performed under the OCP condition of the coin cells after fully discharged. Additionally, the inset provides an enlarged portion of the 0.5 and 2 wt % VC containing electrolyte data. Detailed values for the resistance components can be found in Table 1. Specifically, *R*_b_ denotes the bulk resistance of the cell (electrolyte, separator, and current collector), *R*_EEI_ and CPE_EEI_ signify the resistance and capacitance of the interface layer, *R*_ct_ and CPE_electrode_ represent the charge-transfer resistance and double-layer capacitance, and *W*_warburg_ accounts for the diffusion effect of lithium-ion on the electrodes.^1,27,48^

To gain initial insights into the oxidation pathway of VC, CV experiments were conducted at 0.1 mV/s for the first 3 cycles in coin cells. Prior studies have reported that VC undergoes oxidation at a voltage earlier than that of conventional EC/DEC solvents (above 5.2 V). Oxidation leads to polymerization via reactions between VC molecules in the electrolyte.^27^ As illustrated in Figure 1a,b, VC exhibits oxidation features around 4.252 V during the first three anodic cycles, the current change in this potential range is associated with the VC oxidation and polymerization, aligning with literature.^27^ Interestingly, this oxidation peak is conspicuously absent from the baseline electrolyte. The VC oxidation features coincide with the phase transition range from the second hexagonal (H2) to the third hexagonal (H3) phase, while the oxidation voltage window for EC is above 5.2 V.^27^ Both Figure 1a,b exhibit the leftward-shifted anodic peaks (Ni^2+^/Ni^4+^) at 4.133 and 4.044 V, respectively, from the first cycle to subsequent cycles, implying the reduced polarization of the cathode after the first scan.

Furthermore, the VC-containing electrolyte demonstrates a diminished differential potential (Δ*P*) compared to the baseline electrolyte during CV from 0.559 to 0.310 V. This suggests more efficient kinetics that are associated with lithium-ion mobility in the VC additive electrolyte. These observations suggest that VC undergoes oxidation at an earlier stage in LIBs relative to EC/DEC, thereby initiating a distinct CEI formation pathway compared to the conventional oxidation of EC/DEC. The modified CEI contributes to stabilizing the cathode surface and facilitating ion transportation, leading to reduced surface reconstruction, as detailed in the subsequent section. Notably, the CV curves for the 0.5 wt % VC additive electrolyte did not show a significant change compared to the baseline electrolyte. This observation is likely attributable to only a trace amount of VC present in the electrolyte.

Figure 1c–g presents the rate capability, constant current cycling performance, and the specific charge/discharge curves for different electrolyte systems in coin cells with a cathode loading around 12 mg/cm^2^ [areal theoretical capacity around 3.28 mA h/cm^2^ (ref (44))]. These cells include baseline electrolyte and electrolytes with various concentrations of VC to emphasize the positive effects of VC additives on the performance stabilization and capacity retention of LIBs. The cells underwent 50 cycles in a series of *C*-rate tests ranging from *C*/10 to *C*/2 and back to *C*/10, between 2.8 and 4.3 V. The 2 wt % VC containing electrolyte significantly improved capacity recovery compared to both the baseline and the 0.5 wt % VC electrolytes during the last *C*/10 rate cycles, indicating better stress-sustained ability at high *C*-rates. Nevertheless, the 0.5 wt % VC electrolyte still demonstrated better capacity recovery than the baseline. A similar observation was made in the constant-current cycling test, as shown in Figure 1d; the charge capacity in the baseline electrolyte started to decay after 40 cycles, reaching 167.93 mA h/g_NMC_ with a CE of 90.8% at the 80th cycle. In contrast, the VC containing electrolytes maintained similar capacity to the initial cycles, ending with 176.16 mA h/g_NMC_ with a CE of 96.5% for 0.5 wt % VC and 179.16 mA h/g_NMC_ with a CE of 98.7% for 2 wt % VC.

Figure 1e–g displays the corresponding charge/discharge curves from Figure 1d. The baseline electrolyte exhibited the largest capacity decay in both charge and discharge processes at the 80th cycle, while the VC containing electrolyte showed a superior capacity retention. Moreover, the baseline electrolyte displayed a larger overpotential in the charge process and larger infrared drop in the discharge process at the 80th cycle compared to the other two electrolytes. This is attributed to the leftover lithium ions in the active materials resulting from a lower charge transfer efficiency. In contrast, the VC containing electrolytes both showed only slight capacity changes in the first 20 cycles and much less capacity decay at the 80th cycle compared to the baseline. The 2 wt % VC containing electrolyte also exhibited a smaller overpotential from the first to 80th cycle, indicating a better charge transfer efficiency in this electrolyte system. Notably, in both rate capability test and constant current cycling test, the initial one or two cycles always exhibited higher charge capacity and lower CE compared to the subsequent cycles due to the parasitic reactions and CEI/SEI formation at the beginning of the cycling procedure.

The EIS method was also employed to examine the interfacial resistance and charge transfer resistance after 100 constant-current cycles for the baseline electrolyte, 0.5 wt % VC containing electrolyte, and 2 wt % VC containing electrolyte, respectively. The EIS data along with an equivalent circuit model (ECM) and fitting were utilized to analyze the resistance components and are depicted in Figure 1h. The fitting results for each resistance component are summarized in Table 1. These findings provide compelling evidence that the 0.5 wt % VC additive electrolyte effectively reduces the charge transfer resistance (*R*_ct_) after prolonged cycles from around 810.48 to 33.61 ohms/cm^2^, primarily due to the mitigation of rock-salt formation in comparison to the baseline electrolyte. Notably, the 2 wt % VC containing electrolyte exhibited a slightly higher charge transfer resistance than the 0.5 wt % VC electrolyte, potentially attributed to a thicker CEI/SEI formation on the electrodes owing to the higher concentration of VC. Interestingly, the baseline electrolyte demonstrated the lowest interfacial layer resistance of 53.88 ohms/cm^2^ compared to the electrolytes with VC additives. Furthermore, the electrolyte with 0.5 wt % VC exhibited a lower interfacial layer resistance of 68.9 ohms/cm^2^ than the one with 2 wt % VC of 72.96 ohms/cm^2^. This distinction may be attributed to the formation of a less porous CEI/SEI layer, resulting from VC polymerization on the electrodes. Such a layer increases the interfacial impedance by providing greater insulation to electrons, restricting their mobility through the interface. The thicker CEI/SEI formed with a higher concentration of VC will not only contribute to a higher charge transfer resistance but also result in a higher interfacial impedance. This finding aligns with the results reported by Song et al. regarding changes in interfacial properties.^45−47^ Nonetheless, both VC containing electrolytes demonstrate significantly lower charge transfer resistance compared to the baseline, pointing toward the suppression of rock-salt formation on the NMC cathodes.

**Table 1:** Summary of EIS Fitting Results for Different Resistance Components of Baseline and VC Containing Electrolytes^a^

electrolyte	*R*_b_ (Ω/cm^2^)	*R*_EEI_ (Ω/cm^2^)	*R*_ct_ (Ω/cm^2^)	*X*^2^
baseline	13.13 ± 1.88	53.88 ± 4.23	810.48 ± 70.06^b^	0.009
0.5 wt % VC	16.22 ± 1.79	68.90 ± 7.50	33.61 ± 3.87	0.019
2 wt % VC	15.39 ± 1.58	72.96 ± 6.49	49.72 ± 6.17	0.036

a The table includes the following resistance components: *R*_b_ represents the bulk resistance of the cell comprising the electrolyte, separator, and current collector; *R*_EEI_ represents the resistance of the electrode electrolyte interface layer; *R*_ct_ represents the charge-transfer resistance; and *X*^2^ represents the chi-square statistic of the fitting results. All values are normalized to their respective areal resistance values.

b A similar magnitude of *R*_ct_ was also reported for the baseline electrolyte after prolonged cycles.^49^

**Figure 2:**
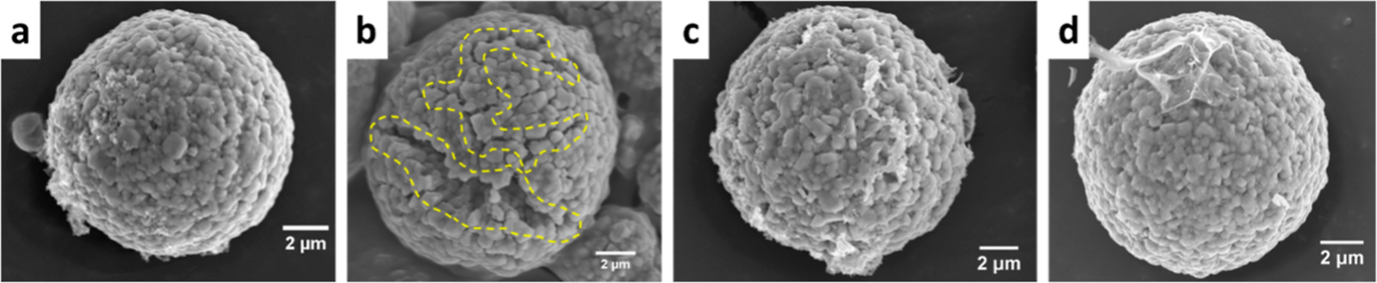
SEM images of an individual NMC particle in (a) its pristine state, (b) after undergoing 100 constant-current cycles with the baseline electrolyte, (c) with 0.5 wt % VC containing electrolyte, and (d) with 2 wt % VC containing electrolyte.

**Figure 3:**
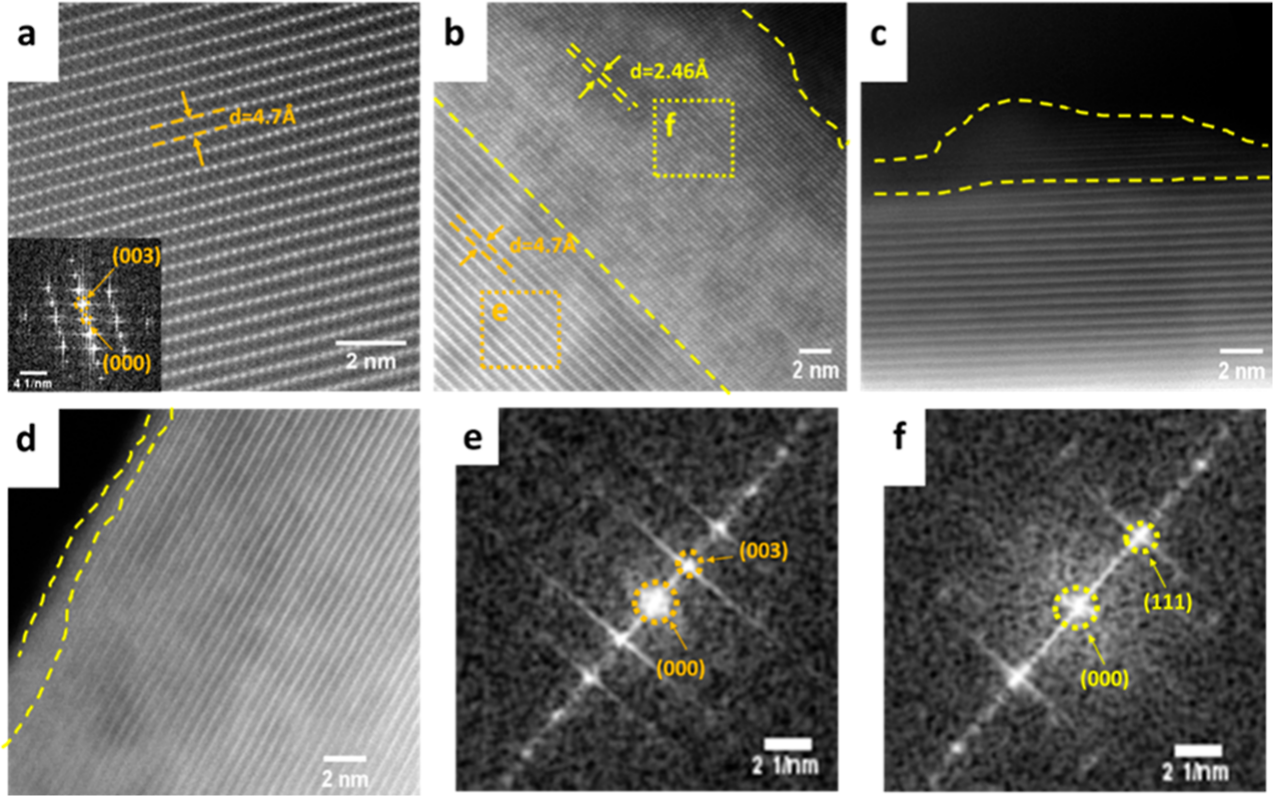
HAADF-STEM images of NMC particles in (a) its pristine state, along with corresponding fast Fourier transformation (FFT) patterns in the inset; after undergoing 100 constant-current cycles with (b) the baseline electrolyte, (c) with 0.5 wt % VC containing electrolyte, and (d) with 2 wt % VC containing electrolyte. The rock-salt layer is indicated by yellow dashed lines. Panels (e,f) present corresponding FFT patterns of the NMC layered structure of space group *R3̅m* indicated in an orange box in panel (b) and rock-salt reconstruction of the *Fm3̅m* space group indicated in a yellow box of panel (b), respectively. Notably, the thickness of the rock-salt layers depicted in panels (c) of 0.5 wt % VC containing electrolyte and (d) of 2 wt % VC containing electrolyte is reduced by up to 80% in comparison to panel (b) under the baseline condition.

SEM analysis of pristine NMC particles and harvested cathodes after 100 constant-current cycles (Figure 2a–d) confirms the efficacy of the VC additive in preserving the morphology of NMC particles, presenting a more intact structure in the VC containing the electrolyte system. Cathodes harvested in the baseline electrolyte exhibit inhomogeneous features with cracks on the particle surface after extended cycles as indicated in the yellow dash lines. For a comprehensive overview, SEM images with a higher particle count for each electrolyte system can be found in the Supporting Information (Figure S2). TEM results (Figure 3a–f) further confirm that VC containing electrolytes offer protection against rock-salt formation on the cathode surface, while the baseline cathodes exhibit a significant region of surface reconstruction, where the pathway for Li^+^ ions is obstructed. Figure S3 displays the lower magnification TEM images, presenting a comprehensive comparison of larger areas between pristine and cycled cathodes as well as baseline and VC containing electrolytes. These findings preliminarily demonstrate that the VC containing electrolytes effectively mitigate the capacity decay of NMC cathodes, maintaining their homogeneity and integrity, and thus resulting in reduced rock-salt formation on the surface.

### 3.2 In Situ Raman Characterization of CEI Formation

**Figure 4:**
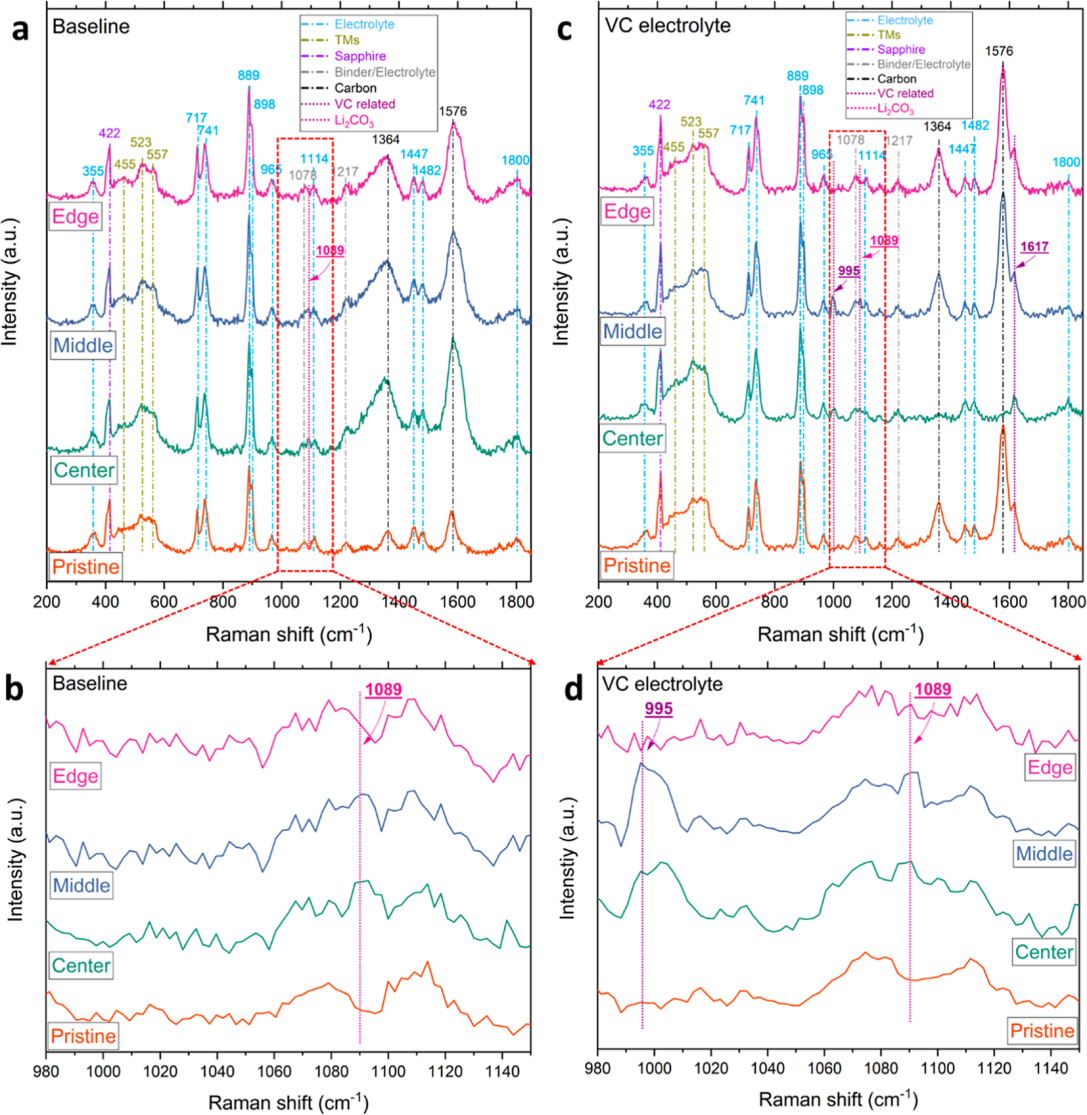
Normalized in situ Raman spectra of pristine cathode and after formation cycles with prominent peaks labeled. Panel (a) shows the in situ Raman spectra of the baseline electrolyte in its pristine condition and after formation cycles at three different locations. Panel (b) provides a magnified view of the range between 980 to 1150 cm^–1^ for the baseline electrolyte. Panel (c) presents the in situ Raman spectra of the 2 wt % VC containing electrolyte in its pristine condition and after formation cycles at three different locations, while panel (d) offers a magnified view of the range between 980 to 1150 cm^–1^ for the 2 wt % VC containing electrolyte.

**Figure 5:**
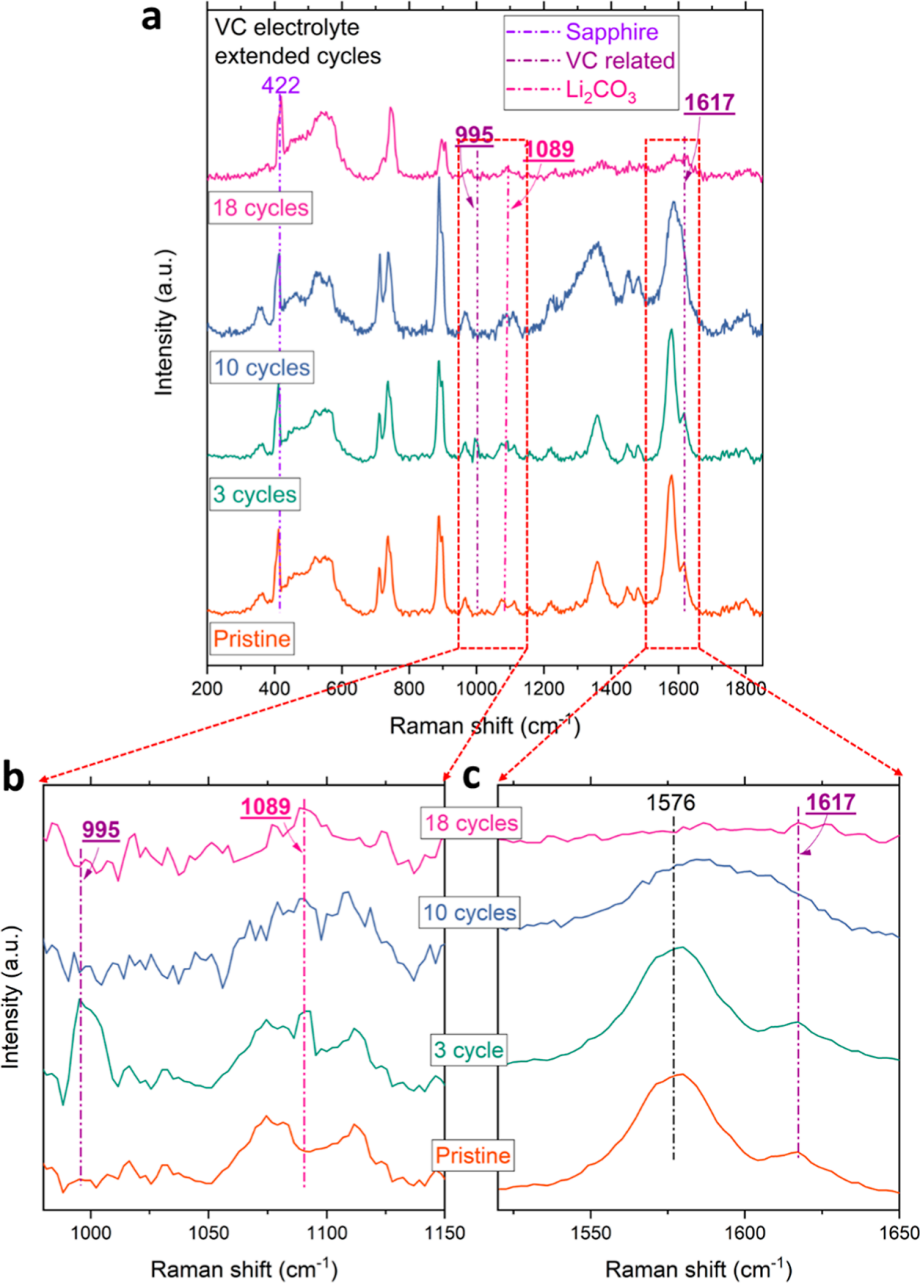
Normalized in situ Raman spectra of the pristine cathode and after extended cycles (up to 18 cycles) in 2 wt % VC containing electrolyte with major peaks labeled. Panel (a) illustrates its pristine condition to extended cycles. Magnified views of specific ranges are presented in panels (b,c) for 980 to 1150 cm^–1^ and 1520 to 1650 cm^–1^, respectively.

To investigate the formation pathway of the modified CEI in both the baseline electrolyte and the VC electrolyte, a series of in situ Raman experiments were conducted. Initially, the Raman cell was subjected to its three formation cycles within a normal voltage range of 2.8 to 4.3 V. Subsequently, the Raman cell was placed under Raman microscopy for in situ surface characterization. The cycling performance of the Raman cell, ensuring the consistency and reliability of the experimental setup, is presented in Figure S4. The in situ Raman results, illustrating the pristine state and the postformation cycles with each major peak appropriately labeled, are shown in Figure 4a–d. To comprehensively cover the spectral range of the transition metal and polymer regions, the Raman spectra were collected within a wavenumber range of 200 to 1850 cm^–1^. Additionally, the range from 980 to 1150 cm^–1^ was magnified and is presented in Figure 4b,d. After the formation cycles, Raman spectra were collected at three locations on the cathode surface from the center to edge (Figure S5) to compare the cathode surface chemistry evolution between the baseline and VC electrolyte at its OCP condition. Additional Raman spectra were collected at extended cycle numbers (up to 18 cycles) to confirm whether the CEI layer continued to grow over longer cycles, as shown in Figure 5a–c.

The Raman spectra from Figure 4a–d exhibited a persistent peak at 422 cm^–1^, which originated from the Al_2_O_3_ corundum material that composes the sapphire optical window of the Raman cell. This peak corresponds to the symmetric stretching (E_g_ mode) of the Al–O bonds in the aluminum oxide lattice and serves as a distinct characteristic feature of the crystal structure of Al_2_O_3_ corundum, as previously reported.^50−52^ Furthermore, this peak was confirmed by comparing the Raman spectra between the NMC cathode with and without a sapphire window in their pristine state, as shown in Figure S6. Thus, to ensure precise data interpretation, we opted to normalize all of the in situ Raman spectra to the peak at 422 cm^–1^, a procedure also applied to the operando Raman spectra in a later section. This decision was made because the cell was stationary during in situ/operando Raman acquisitions, and the geometric configuration between the electrodes and sapphire window remained constant. Therefore, the distinctive peaks of the sapphire window can serve as a dependable and constant parameter rather than a mere interference.

The in situ Raman spectra of both the baseline (Figure 4a,b) and VC electrolyte (Figure 4c,d) shared common peak features, each corresponding to specific molecular vibrations and components. The peaks at 455, 523, and 557 cm^–1^ are attributed to the in-plane Ni–O bend mode, in-plane Co–O bend mode, and out-of-plane Ni–O stretch mode, respectively, representing the transition metal region (indicated by yellow dash lines). The peaks at 717 and 889 cm^–1^ correspond to the out-of-plane ring bending mode and symmetrical ring breathing mode of EC, while the peak at 741 cm^–1^ relates to the symmetrical vibration mode of PF_6_^–^, and the 898 cm^–1^ peak is attributed to the symmetrical ring breathing mode of Li^+^ associated with EC (Li^+^–EC), representative of the electrolyte region (indicated by blue dash lines). Additionally, the two prominent peaks at 1364 and 1576 cm^–1^ arise from the carbon D and G bands, respectively (indicated by black dashed lines), introduced by conductive carbon during electrode fabrication. Notably, a new peak at 1089 cm^–1^ emerges after the formation cycles (Figure 4a–d) and persists in prolonged cycles (Figure 5a–c), identified as Li_2_CO_3_, a major component of the CEI (indicated by pink dash lines).

Noteworthy is a new peak that merged in the spectrum of 2 wt % VC containing electrolyte at 1617 cm^–1^ (rightmost purple dash line in Figure 4c). It can be attributed to the C=C double bond stretching mode of VC, an indication that VC is indeed present. An intriguing discovery is the emergence of a previously unidentified peak at approximately 995 cm^–1^ on specific regions of the cathode surface. This peak is indicated by the leftmost purple dashed lines in Figure 4b and the sole purple dashed line in Figure 4d. Importantly, this peak appears after the formation cycles and is absent in both the pristine state and the postformation cycles of the baseline electrolyte, as shown in Figure 4a,c. This could be a result of the C–O stretching modes of semi-carbonates that are related to the radical polymerization of VC triggered after oxidation.^27,31,34,53,54^ It is clear from this evidence that VC has been oxidized during the formation cycles and polymerized to form the modified CEI layer. On the other hand, it is important to note that the characteristic peak observed at 995 cm^–1^ on the cathode surface was not present at all locations due to variations in the current distribution on the cathode surface, as indicated in Figure 4b and d. Furthermore, employing a laser spot size below 10 μm, coupled with the intrinsic nonuniform particle distribution and occasional electrolyte interference, led to fluctuations in the intensity of the transition metal region (400–600 cm^–1^), carbon region (1364 and 1576 cm^–1^), as well as variations in the EC intensity (717 and 889 cm^–1^).

Another interesting observation is that at extended cycles (Figure 5a), the peak at around 995 cm^–1^ was difficult to discern after 10 and 18 cycles (Figure 5b). This may be due to the formation of different CEI such as Li_2_CO_3_ or EC oxidation during prolonged cycling, and the Raman scattering associated with VC polymerization could be lost in the newer deposition on the cathode surface. Furthermore, as depicted in Figure 5b,c, the Raman peak at 1617 cm^–1^ was not present in the spectra collected after 10 and 18 cycles. The conspicuous absence of VC signals strongly suggests its consumption during the formation cycles of the early stage CEI layer, which is further corroborated by the emergence of the peak at 995 cm^–1^ in Figure 5a,b, indicating the polymerization of VC after the formation cycles and its depletion in prolonged cycles.

Further analysis reveals minor peaks in Figure 4a,c at 355, 965, 1114, 1447, and 1482 cm^–1^, attributed to background signals originating from the electrolyte (blue dash lines). Additionally, two other peaks at 1078 and 1217 cm^–1^ are attributed to the mutual interaction between the electrolyte and the binder (PVDF) (gray dash lines). The identification of these peaks was validated by analyzing reference samples of pure NMC811 powders, pure NiO powders, pure carbon, LiPF_6_ (EC/DEC) solution, pure PVDF powders, and pure Li_2_CO_3_ powders, as shown in Figure S7. The determination of the mutual interaction peak was achieved through a comparison of the Raman spectra of the LiPF_6_ (EC/DEC) solution and PVDF powders. The identification of Raman peaks was summarized in Table S1.

### 3.3 Operando Raman Characterization of Rock-Salt Formation

**Figure 6:**
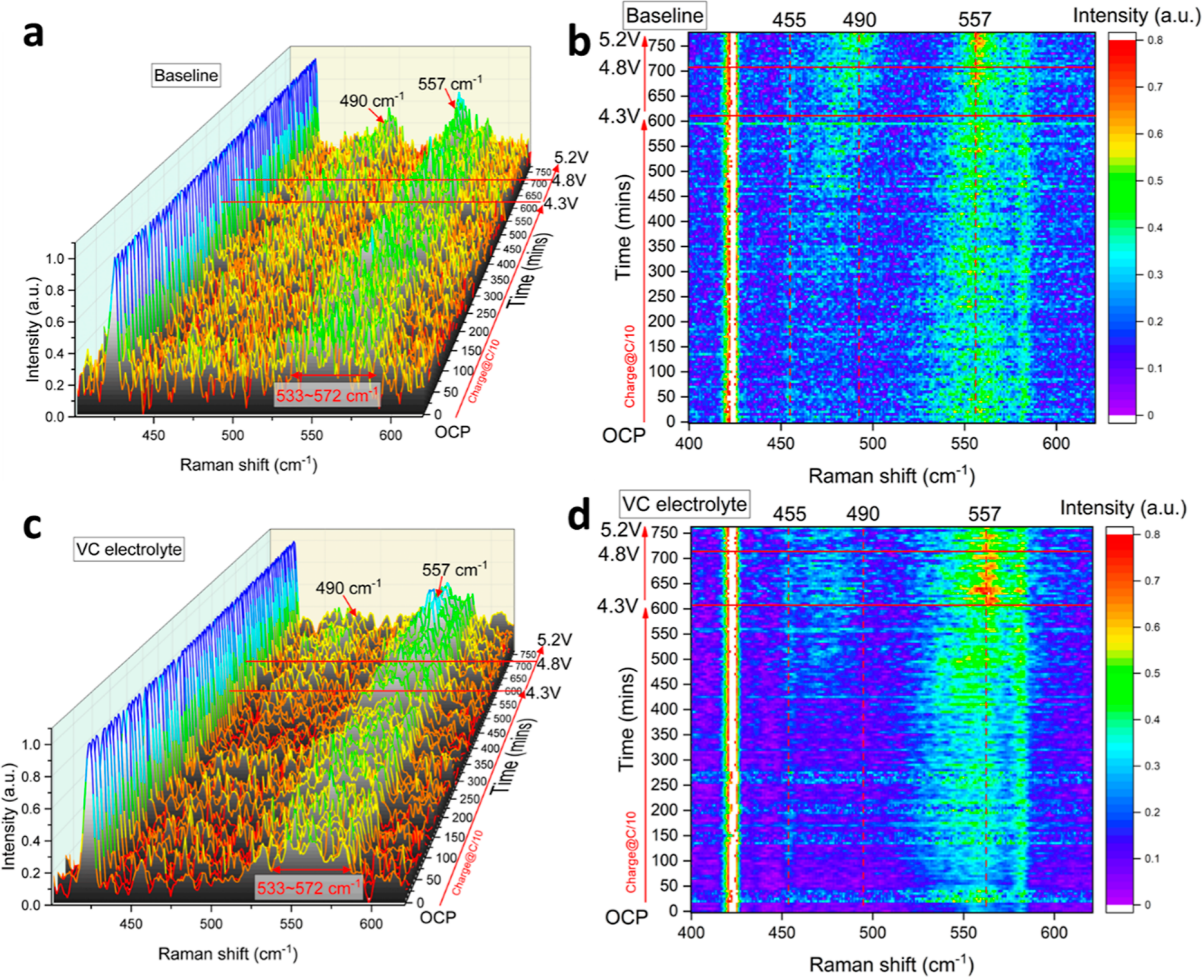
Normalized operando Raman spectra during the charge process up to 5.2 V. Formation cycles were first performed before this charge to 5.2 V. Panel (a) depicts the baseline electrolyte, and panel (b) presents the corresponding intensity heat map on a scale from 0 to 0.8 for enhanced visualization. Panel (c) illustrates the 2 wt % VC containing electrolyte, and panel (d) showcases its corresponding intensity heat map on a scale from 0 to 0.8 for enhanced visualization.

In this section, we embarked on an investigation of the operando Raman spectra evolution of the baseline electrolyte (1 M LiPF_6_ in EC/DEC) and VC electrolyte (1 M LiPF_6_ in EC/DEC + 2 wt % VC) during the initial charge process after the optical cell completed its formation cycles at normal operation voltage window between 2.8 and 4.3 V. The measurements were performed from its OCP to an ultrahigh voltage scenario of 5.2 V. The operando Raman spectra and their corresponding intensity heat map are depicted in Figure 6. The Raman shift was acquired in the range of 400 and 620 cm^–1^, where most of the transition metal interactions were anticipated, as previously reported.^40,55,56^ The operando Raman spectra exhibited two persistent peaks at 422 and 578 cm^–1^, which correspond to the symmetric stretching (E_g_ mode) and the bending mode (A_1g_ mode) of the Al–O bonds in the aluminum oxide lattice, respectively. Normalization of all spectra to the 422 cm^–1^ peak was carried out, following the procedure used in the previous CEI characterization section. For enhanced visualization of the transition metal range, the scale of the heat map shown in Figure 6b,d was set from 0 to 0.8 to magnify the changes, while the scale used in the operando Raman spectra presented in Figure 6a,c remained unchanged, ranging from 0 to 1.

The most intriguing observation from Figure 6a,b is that, during the ultrahigh voltage range of 4.3 to 5.2 V, a new peak appears around 490 cm^–1^, visible both in the operando spectra and more clearly in the intensity heat map. This could be explained by the one-phonon longitudinal optic mode (1LO mode) of NiO on the surface of charged NMC811, indicating the ongoing formation of rock-salt NiO as previously reported,^40,57^ which was also consistent with our ex situ Raman spectroscopy of pure NiO displayed in Figure S7. The appearance of NiO was a result of the oxygen loss and Ni^+^/Li^+^ mixing phenomenon on the surface of charged NMC811, which occurred when the voltage approached the upper cutoff voltage of 4.3 V and above, as demonstrated in the scenario in the present work. It is generally accepted that the ultrahigh operating voltage can accelerate the formation of rock-salt in NMC cathodes, in addition to the effect of prolonged cycle numbers.^40,57^ This proposition was further substantiated through our TEM analysis, which revealed the presence of a thick rock-salt layer in an NMC811 particle subjected to 100 cycles within a normal operating voltage range of 2.8 to 4.3 V. In contrast, the thickness of the rock-salt layer in the other VC containing electrolytes was significantly lower by roughly 80%, as illustrated in Figures 3 and S3. The corresponding FFT patterns of the rhombohedral region, indicated by the orange box in Figure 3b, and the rock-salt region, indicated by the yellow box in Figure 3b, are presented in Figure 3e,f, respectively. The *d*-spacing along the (003) plane, highlighted in orange for both the pristine NMC (*R3̅m* space group) and cycled NMC (Figure 3a,b), aligns with the literature.^58^ Similarly, the *d*-spacing along the (111) plane, highlighted in yellow for the cycled NMC in Figure 3b (*Fm3̅m* space group), aligns with the literature.^58,59^

We further conducted the operando Raman spectra acquisition on the 2 wt % VC containing electrolyte, as shown in Figure 6c, and the corresponding intensity heat map is shown in Figure 6d. Except for the 490 cm^–1^ range, which originates from the one-phonon longitudinal optic mode (1LO mode) of NiO, the Raman shift evolution pattern was found to be similar. After 450 min, the intensity in this range was significantly lower than the baseline at the same state of charge (SoC), where the formation of NiO rock-salt was observed in the baseline spectra. These results clearly suggest that the NiO rock-salt formation was less severe in the 2 wt % VC containing electrolyte system than in the baseline electrolyte, indicating the well-preserved NMC layered structure under the ultrahigh operation voltage scenarios. In order to obtain a more comprehensive understanding of the Raman spectra evolution, we have compared the specific Raman spectra for the two different electrolytes at various operating voltages in Figure S8. It becomes evident that the peak intensity at 490 cm^–1^ shows a significant increase at and after 4.6 V in the baseline electrolyte, whereas such an increment is absent in the 2 wt % VC containing electrolytes. It is important to highlight in these experiments that the charging conditions were deliberately designed to accelerate the surface reconstruction process, which typically occurs at high operation voltages or after an extended number of cycles. These conditions were designated to simulate cell abuse. Therefore, it is reasonable to infer that the VC additive would operate through a similar mechanism to prevent the formation of rock-salt layers during extended cycles, as supported by the TEM results in Figures 3 and S3.

In both Figure 6a,c, prior to charging the cell to 4.3 V, a broad peak ranging from 533 to 572 cm^–1^ was persistently detected in the operando Raman spectra. As the voltage was increased to 5.2 V, the intensity of this peak increased, as shown in Figure 6a–d. This peak is attributed to the layered structure of the NMC cathode and is associated with the Raman active modes of most transition metal oxides, consistent with the observations in the CEI characterization section (Figure 4a–d). A broad peak at 557 cm^–1^ observed in both baseline and VC containing electrolytes is attributed to the bending mode of the Ni–O interaction from the *R̅*3*m* space group, while the symmetric stretching of Ni–O, Co–O, and Mn–O at 455, 518, and 595 cm^–1^, respectively, as well as the bending mode of Co–O and Mn–O at 534 and 611 cm^–1^, are also expected to contribute to this range, resulting in an even broader peak. However, the operando measurements in this study faced challenges in adequately capturing the Co–O and Mn–O interactions due to the utilization of a Ni-rich type of layered NMC811 cathode, exhibiting an approximate 80% Ni molar ratio. The gradual increase in the intensity of the broad peak at 557 cm^–1^ can be attributed to a significant contraction in the crystal structure resulting from overdelithiation under the extreme voltage conditions. Nevertheless, it should be noted that the broad peak ranging from 533 to 572 cm^–1^ is a combined contribution from the bending mode of Ni–O and Co–O. Additionally, the 455 cm^–1^ peak may not be clearly observed in the operando spectra of Figure 6a,c. However, the corresponding pattern in this range (455 cm^–1^) and the prominent peak at 557 cm^–1^ remain evident in the corresponding intensity heat map of Figure 6b,d. These observations collectively indicate the prevalence of Ni–O interactions in the electrodes.

At this point, we observed a causal relationship between the modified CEI layer and the inhibition of the rock-salt phase in NMC materials. The more cohesive CEI layer, a result of VC polymerization during the initial formation cycles, aids in the active material’s resilience to mechanical strain caused by anisotropic lattice volume changes during cycling. This preservation of more intact morphological characteristics is confirmed by our SEM data in Figure 2. It is widely acknowledged that ultrahigh operating voltage can expedite the formation of rock-salt in NMC cathodes, compounded by the effects of prolonged cycling.^40,57^ In our study, the more homogeneous and less porous CEI layer also facilitates lithium-ion mobility between the NMC surface and electrolyte, thereby reducing lithium-ion and nickel-ion mixing induced by the stress of lithium-ion extraction and insertion, especially under high operating voltage scenarios. Surface rock-salt reconstruction is therefore inhibited.

### 3.4 In Situ XAS Characterization of the Ni–O Bond

In situ XAS measurements share several advantages with Raman spectroscopy, such as minimal interference and contamination of electrochemical processes, the ability to monitor local structural changes in real-time, and the capability to investigate fast-transient or nonequilibrium reactions at high resolution. Specifically, X-ray absorption near-edge structure (XANES) analysis provides valuable information about both the geometric and electronic structures of the studied system, while extended X-ray absorption fine structure (EXAFS) analysis provides additional local structural information around the central atom, allowing for a quantitative comparison between experimental results and theoretical models. Therefore, in order to assess the effect of VC on rock-salt formation more accurately, two sets of in situ cells were established: formation cells and cycled cells. The formation cells were fabricated under pristine conditions and underwent 3 cycles at the synchrotron facility, whereas the cycled cells had been subjected to 20 cycles prior to the XAS measurements and were prepared in the laboratory. XAS spectra were acquired for both the baseline and 2 wt % VC containing electrolyte systems at the OCP state, 4.3 and 4.8 V.

**Figure 7:**
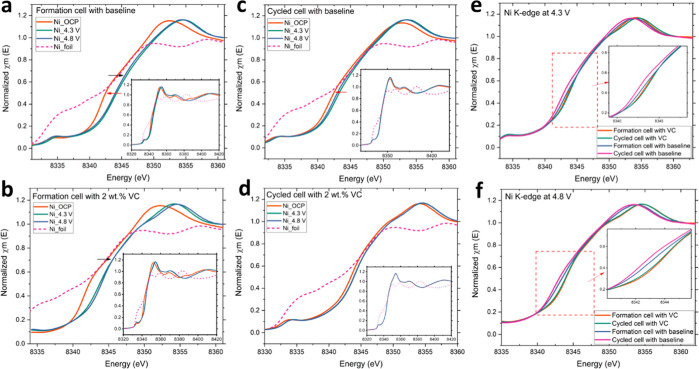
Normalized in situ XANES spectra at Ni K-edge during the initial charge process of the two sets in situ XAS cells: after formation cycles (a,b) and extended cycles (c,d), with Ni^0^ foil as a reference. Panels (a,b) depict the XANES of Ni after formation cycles in the baseline and 2 wt % VC containing electrolytes, respectively. Panels (c,d) show the XANES of Ni after prolonged cycles in the baseline and 2 wt % VC containing electrolytes, respectively. Panels (e,f) provide a comprehensive summary of the XANES of Ni K-edge in both sets of XAS cells (formation and cycled) for the two electrolyte systems at 4.3 and 4.8 V, respectively. The insets show the full range of the normalized XAS spectra for panels (a–d) and the magnification of edge shift in XAS spectra for panels (e,f).

We first scrutinized the formation cells, and Figure 7a,b shows that there was a noticeable shift in the Ni K-edge from its OCP to a higher cutoff voltage of 4.3 V, suggesting the oxidation of the Ni metal center.^60,61^ In contrast, the Co and Mn K-edges remained relatively unchanged during the charging process (Figures S9 and S10). This indicates that Ni is the predominant element involved in the electrochemical process and is the main capacity contributor for this type of NMC, aligning with our predictions. While Co and Mn were inactive during most of the cycle, their local structure was observed to change based on the shape change from XANES, consistent with previously reported results.^60,61^ A further interesting observation is that, at the ultrahigh operation voltage of 4.8 V, the Ni K-edge surprisingly exhibited a slight leftward shift in the baseline electrolyte (blue line in Figure 7a). This shift is primarily attributed to the surface reconstruction of NMC: the mixing of Ni^+^ and Li^+^ led to the formation of a newly generated NiO layer on the surface that reduced the overall Ni oxidation state and subsequently shifted the edge position.^12^ Surface reconstruction is accelerated when the cutoff voltage was increased to 4.8 V, resulting in a more pronounced edge shift. In contrast, the Ni K-edge position of the VC containing electrolyte (Figure 7b) at voltages of 4.3 and 4.8 V remains virtually unchanged, indicating that the VC effectively suppressed reconstruction on the NMC cathode surface.

The normalized XANES spectra of cycled cells are illustrated in Figure 7c,d. Similarly, the cell containing baseline electrolyte showed a minor left shift at 4.8 V in comparison to 4.3 V, indicating the formation of rock-salt in a VC-free environment after 20 cycles. The VC containing the electrolyte system revealed a more stable Ni oxidation state, with edge positions almost indistinguishable at 4.3 and 4.8 V, suggesting less rock-salt formation on the surface. All cells exhibited a right edge shift from their OCP to a higher voltage due to the valence state increase of Ni, consistent with that observed in formation cells. However, this rightward shift was less significant than that in formation cells, as anticipated, since a certain amount of charge capacity was lost during previous cycles. This observation aligns with the findings of Tallman et al., albeit a moderate edge shift was reported in their work.^60^

The normalized in situ XANES spectra at 4.3 and 4.8 V were further compared to investigate the differences in Ni oxidation state between formation and cycled cells, as shown in Figure 7e,f. The results showed that in baseline cells (blue and purple lines in Figure 7e,f), Ni exhibited the largest deviation of edge position among formation cells and cycled cells at both voltages, indicating a deeper rock-salt phase was responsible for the more intense Ni reduction behavior of the NMC cathode. However, the edge positions of the 2 wt % VC containing electrolytes (red and green lines in Figure 7e,f) were almost overlapped on the right side of baseline edges, indicating a lower Ni reduction resulting from shallower rock-salt phase formation on the NMC cathode surface. This comparison of Ni oxidation state at the same operation voltage provides compelling evidence that the VC additive positively contributed to the suppression of rock-salt formation on the cathode.

### 3.5 Differential Radial Distance Analysis of the Ni–O Bond from FT-EXAFS

Layered insertion materials like NMC cathodes undergo only minor structural changes during cycling, as they facilitate the intercalation and deintercalation of lithium ions in the electrode. However, the rock-salt layer in NMC particles is generally considered to be an extremely thin layer which is also confirmed by our TEM data in Figures 3 and S3, and there have been some concerns about the accuracy of hard X-ray measurements, which are bulk-sensitive characterization methods.^10,60,61^ To overcome this, a more detailed analysis through EXAFS modeling provides ample information about the local structure that occurs at Ni K-edges.

**Figure 8:**
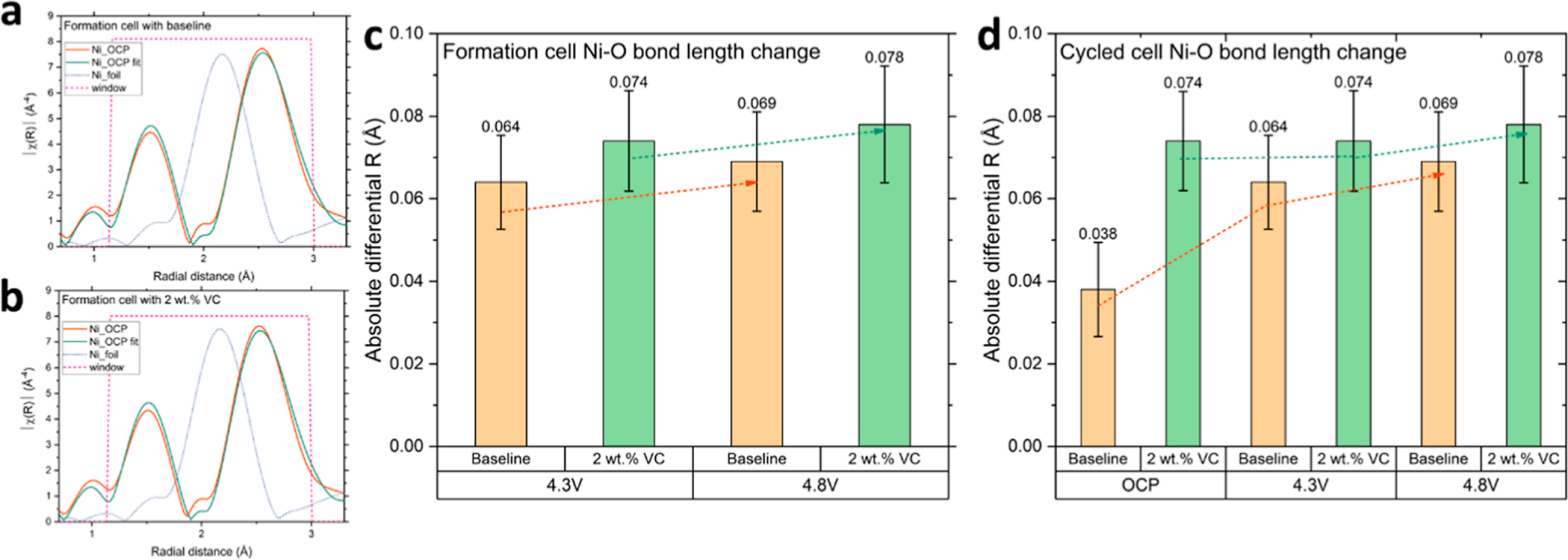
Ni K-edge FT-EXAFS spectra (a,b) with corresponding fitting results, Ni^0^ foil reference, and fitting window, as well as the differential *R*-value of Ni–O bond length analysis results (c,d). Panel (a) showcases the spectra at the OCP conditions for the baseline electrolyte after formation cycles, whereas panel (b) illustrates the corresponding spectra for the 2 wt % VC containing electrolyte after formation cycles. Panel (c) exhibits the differential *R*-value of the Ni–O bond length in the formation cells at different voltages compared to their OCP conditions, and panel (d) displays the differential *R*-value of the Ni–O bond length in the cycled cells compared to the OCP condition of the formation cells.

In order to ensure the reliability of the fitting model and results, the EXAFS spectra at the Ni K-edge were analyzed for the initial state condition (OCP) of the baseline electrolyte and 2 wt % VC containing electrolytes. The corresponding quantitative fitting results and FT-EXAFS spectra (Fourier transform of EXAFS spectra) revealed that there were only negligible differences in the Ni–O and Ni–Ni bond lengths in the two measurements, as shown in Figure 8a,b and Table 2. Therefore, the Ni–O bond length in the OCP condition of NMC cathodes, which ranges from 1.94 to 1.95 Å, was adopted as the baseline for subsequent differential radial distance (*R*) analysis with the *R3̅m* space group of the NMC framework. On the other hand, the average Ni–O bond length in the rock-salt structure of NiO framework (*Fm3̅m* space group) was approximately 2.09 to 2.11 Å, indicating a marked difference in Ni–O bond length between the two crystal structures.^61,62^ Notably, the Ni–O bond length in NMC cathodes at its OCP obtained from our fitting results is consistent with the literature,^61,62^ further validating the accuracy of our measurements and model fitting. It should be noted that the measurements were 0.4 to 0.5 Å less than the actual atomic distances determined by FT-EXAFS due to the phase shifts during the photoelectron scattering process.

**Table 2:** Summaries of FT-EXAFS Fitting Results of Ni at the K-Edge (Figure 8a,b) at the OCP Condition^a^

figure index	scattering	*R* (Å)	σ^2^ × 10^–3^ (Å^2^)
8a	Ni–O	1.954 ± 0.009	3.7 ± 1.2
	Ni–Ni	2.882 ± 0.006	
8b	Ni–O	1.954 ± 0.010	3.9 ± 1.3
	Ni–Ni	2.882 ± 0.007	

a The FT-EXAFS fitting was done at Ni K-edge in *R*-space, *k*^1,2,3^ weighting, with a window of 1.15 < *R* < 3 Å. The fitting results of *E*_0_ are 1.64 ± 0.33 and 1.59 ± 0.46 eV at OCP for the formation cell and cycled cell, respectively. *S*_0_^2^ was fixed at 0.784 for Ni K-edge obtained by fitting the corresponding reference foil. The values of σ^2^ for the two fitting results closely match each other as they were obtained at the same SoC and had similar OCP during XAS acquisition.

In addition, during the charging process, the Ni–O bond length decreases due to the crystal contraction caused by delithiation, which is observed in the in situ FT-EXAFS results and confirmed by the published work.^61^ Hence, considering the marked distinction in Ni–O bond length between the two crystal structures, it can be inferred that the discrepancy in Ni–O bond length between the OCP and charged state of a given cell would be relatively diminutive if a greater degree of rock-salt formation were to occur. In light of this rationale, we proceeded to generate the plots of all of the FT-EXAFS spectra at the Ni K-edge from both formation and cycled cells at various voltages, along with its corresponding fitting results and Ni reference foil, which are presented in Figure S11. The full quantitative analysis of the specific Ni–O bond length and coordination number was summarized in Table S2. Subsequently, as illustrated in Figure 8c, an extensive FT-EXAFS investigation of formation cells was conducted to examine the absolute value of differential *R* in Ni–O bond length under different voltages compared to its OCP. This was achieved by subtracting the OCP Ni–O bond length from its counterparts at various voltages in formation cells. Additionally, the Ni–O bond length in cycled cells was analyzed by subtracting the OCP Ni–O bond length of 1.945 Å from its OCP to different operating voltages. A distinct pattern emerged from the analysis, showing that the differential *R* in the Ni–O bond was more pronounced in both formation and cycled cells with 2 wt % VC containing electrolytes than in the baseline electrolyte, indicating less rock-salt formation with the presence of VC. Interestingly, the magnitude of the differential *R*-value of Ni–O bond length (green bars) for 2 wt % VC containing electrolytes in Figure 8c,d were found to be in the same range (0.074 to 0.078 Å), whereas in the baseline electrolyte (yellow bars), the values in cycled cells (0.038 Å) were notably smaller than those obtained from formation cells (0.064 Å). The increase trend (green and yellow dash arrow) in both the formation cell and cycled cell, as depicted in Figure 8c,d, from their OCP to 4.3 and 4.8 V is a result of Ni–O bond length change due to crystal contraction during delithiation.^61^ This observation suggests that rock-salt formation was suppressed in the presence of additive electrolytes, whereas it remained growing throughout the multiple cycles in baseline electrolytes.

The operando Raman spectra and in situ XAS spectra effectively identified a more pronounced rock-salt reconstruction on the cathode surface in the baseline electrolyte. This occurrence may be attributed to the formation of a more porous and less cohesive CEI layer on the cathode surface in the baseline electrolyte. As a result, the NMC structure becomes more susceptible to the mixing of lithium- and nickel-ions due to the stress associated with lithium-ion extraction and insertion.

Notably, the absence of a Raman shift peak around 490 cm^–1^ (Figure 6c,d) and a larger differential Ni–O bond length (Figure 8) in the VC additive electrolytes indicated the successful mitigation of rock-salt reconstruction. This observation is further supported by our TEM results, as illustrated in Figure 3. These findings collectively suggest a causal relationship between the enhanced CEI layer and rock-salt reconstruction on the NMC surface.

### 3.6 VC Oxidation and Polymerization Pathway

**Figure 9:**
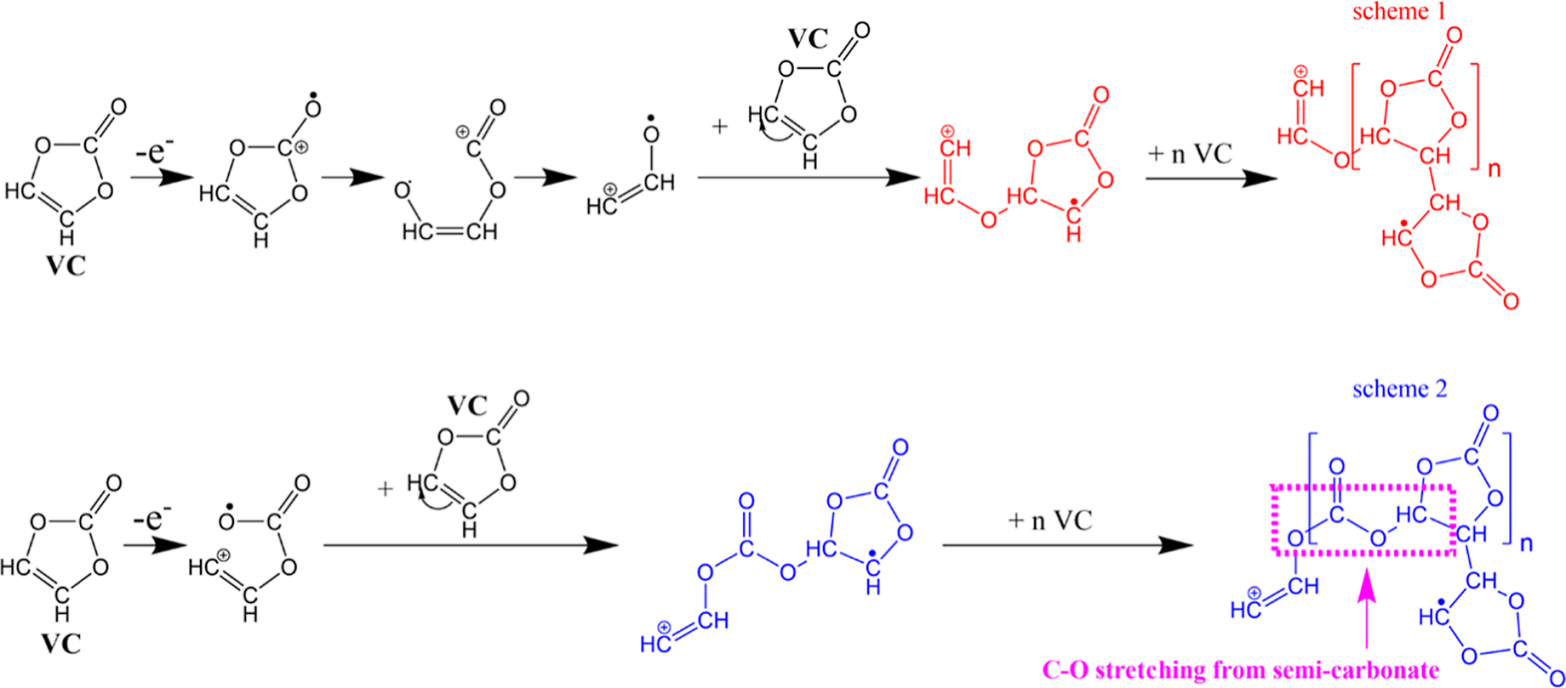
Scheme 1 illustrates the VC oxidation and polymerization pathways based on literature findings from ref (27), used under Creative Commons Attribution Non-Commercial No Derivatives 4.0 License (CC BY-NC-ND, https://creativecommons.org/licenses/by-nc-nd/4.0/); while scheme 2 presents the novel VC oxidation and polymerization pathway proposed in this study. The C–O stretching mode from semi-carbonate is labeled with a purple box.

VC is a widely used electrolyte additive known for its ability to enhance the stability of the CEI and mitigate undesirable side reactions between the electrolyte and the electrode, thereby extending the cycle life of the battery. Previous investigations by Jin et al. have suggested that one of the major products of VC polymerization is branched or cross-linked poly(ethylene oxide) (PEO), while Pritzl et al. proposed a ring-opening mechanism involving the disruption of the C–O bond, as depicted in scheme 1 in Figure 9.^27,32^ However, our in situ Raman spectra revealed the presence of semi-carbonate compounds not accounted for in these mechanisms. Consequently, we propose a slightly different pathway for VC oxidation and polymerization based on our in situ/operando-based experiments and electrochemical measurements, as depicted in scheme 2 in Figure 9.

Schemes 1 and 2 depict distinct yet interconnected pathways involving the ring-opening of VC. However, in the presence of specific reaction conditions, not all VC monomers undergo complete ring-opening and polymerization.^63^ We propose a VC polymerization pathway involving partial ring-opening and interaction with other VC molecules, culminating in the creation of complex entities referred to as semicarbonates. This hypothesis is grounded in the observations derived from in situ Raman spectra during the characterization of the CEI layer, as depicted in scheme 2 in Figure 9. VC initiates the process by losing an electron, leading to the cleavage of (=CH–O−) and the generation of an oxygen radical. This radical then engages in partial ring opening, effectively initiating polymerization with another VC molecule. The semi-carbonates comprise the primary constituents of a modified CEI film. These semi-carbonate-based CEI layers exhibit more polymeric and cohesive characteristics, providing superior protection on NMC cathodes. Therefore, the NMC particles exhibited more intact characteristics, even after prolonged cycles. These conclusions are supported by our SEM images and in situ Raman spectroscopy (Figures 2 and 4). Consequently, the enhanced CEI provides a physical barrier that inhibits destructive side reactions at the cathode surface. It suppresses the formation of rock-salt phases and the Ni^+^/Li^+^ mixing, thus mitigating surface reconstruction. Additionally, this cohesive and less-porous CEI will also protect the NMC particles from the attack by hydrofluoric acid resulting from LiPF_6_ decomposition during prolonged cycling. This conclusion is further substantiated by our TEM images, operando Raman spectroscopy, and in situ XAS analysis (Figures 3 and 6–8).

The CEI derived from VC and other similar additives, with their polymeric and cohesive nature, can better withstand the structural and chemical transformations that occur during cycling while maintaining the integrity of the cathode material. This improved CEI not only enhances the overall performance and capacity retention of LIBs but also offers insight into innovative electrolyte design for next-generation energy storage systems.

## 4 Conclusions

The present investigation delves into the enhanced CEI layer resulting from the impact of VC polymerization and its influence on the suppression of rock-salt formation on NMC cathode surfaces using advanced in situ/operando Raman and in situ XAS techniques, complemented by detailed EXAFS analysis. Initially, we validated that VC undergoes oxidation at an earlier stage than that of EC through CV experiments. The normalized in situ Raman spectroscopy further confirmed the CEI formation pathway arising from VC polymerization during electrochemistry, as indicated by the detection of a new peak at 995 cm^–1^, representing C–O stretching modes resulting from the radical polymerization of VC. SEM images corroborated the distinct morphological features of the NMC particles after numerous cycles.

Furthermore, the normalized operando Raman spectra demonstrated that the presence of the VC additive led to less pronounced rock-salt formation compared to the baseline, evidenced by a significant reduction in the intensity of the Raman peaks corresponding to the 1LO mode of NiO at around 490 cm^–1^ under ultrahigh voltage conditions (above 4.8 V). Additionally, our normalized XANES results indicated a reduced edge shift observed at the Ni K-edge in both formation and cycled cells. A sophisticated EXAFS analysis was performed, which provided further confirmation of VC’s suppressive effect on rock-salt formation by comparing the differential *R* values of Ni–O bond length. TEM results also verified that less Ni^+^/Li^+^ mixing was observed in VC containing electrolyte cells.

Overall, our in situ/operando-based characterization methods successfully demonstrated the CEI formation resulting from VC polymerization and its consequential suppressive effect on surface reconstruction. The experimental findings have led to the formulation of a novel mechanism explaining VC oxidation and polymerization. This study not only underscores the beneficial utilization of electrolyte additives in enhancing cathode performance but also establishes a pioneering approach to unraveling the mechanisms underlying such enhancements, thus guiding future endeavors in the innovative electrolyte design for LIBs.

## Supporting Information Available

Photographs and schematic illustrations of in situ/operando Raman cell and in situ XAS cell, EC performance of Raman cell and XAS cell, SEM images with higher particle count and TEM images with lower magnification, ex situ Raman spectra of all standard references, in situ XANES spectra of Co and Mn K-edge, and FT-EXAFS of in situ XAS cell at different SoC and with all electrolytes (PDF)
