# Supplementary material for: Exploring the Role of an Electrolyte Additive in Suppressing Surface Reconstruction of a Ni-Rich NMC Cathode at Ultrahigh Voltage via Enhanced In Situ and Operando Characterization Methods
Source: ACS Appl Mater Interfaces. 2024 Feb 9;16(7):8639–54. doi: 10.1021/acsami.3c15670 (PMC10895582; doi:10.1021/acsami.3c15670)
Supplement: Supplementary file 1 — am3c15670_si_001.pdf [file am3c15670_si_001.pdf]

Supporting Information for

Exploring the Role of an Electrolyte Additive in Suppressing Surface Reconstruction of Ni-rich NMC Cathode at Ultra-high Voltage via Enhanced In-Situ and Operando Characterization Methods

*Huidong Dai[1], Luisa Gomes[1], Derrick Maxwell[2], Somayeh Zamani[3], Kevin Yang[2], Dianne Atienza[3], Nilesh Dale[3], Sanjeev Mukerjee[1]**

[1]Department of Chemistry and Chemical Biology, Northeastern University, 360 Huntington Avenue, Boston, Massachusetts, 02115, United States

[2]Department of Chemical Engineering, Northeastern University, 360 Huntington Avenue, Boston, Massachusetts, 02115, United States

[3]Nissan Technical Center North America, 39001 Sunrise Drive, Farmington Hills, Michigan, 48331, United States

*Corresponding author: s.mukerjee@northeastern.edu

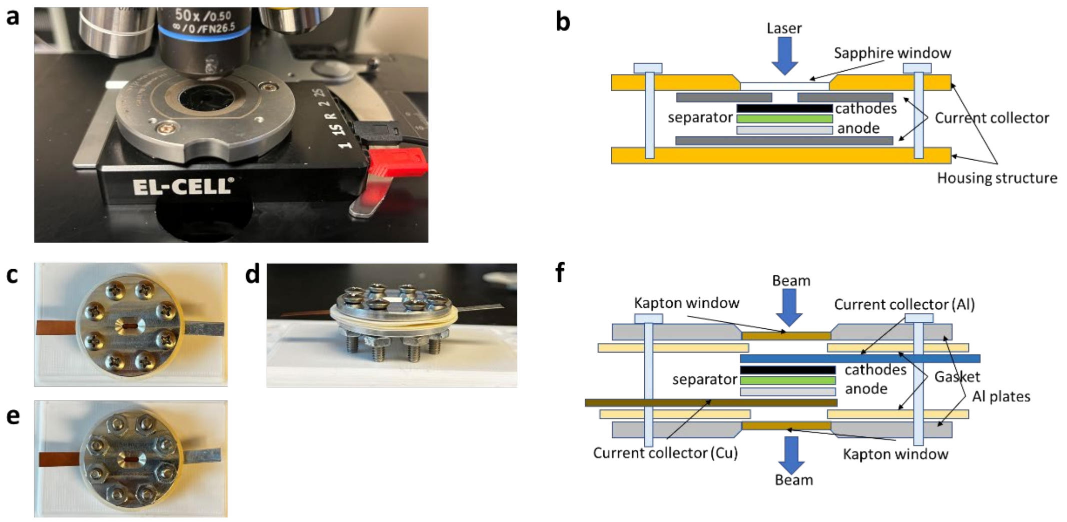

Figure S1 Panel (a) shows photographs of the in-situ/operando Raman cell, also known as El-Cell, the corresponding schematic illustration was shown in panel (b). Panels (c-e) display the in-situ X-ray absorption spectroscopy (XAS) cell that was designed and machined in-house. Specifically, panel (c) provides a top view, panel (d) shows the side view, and panel (e) displays the bottom view. The corresponding schematic illustration is shown in panel (f).

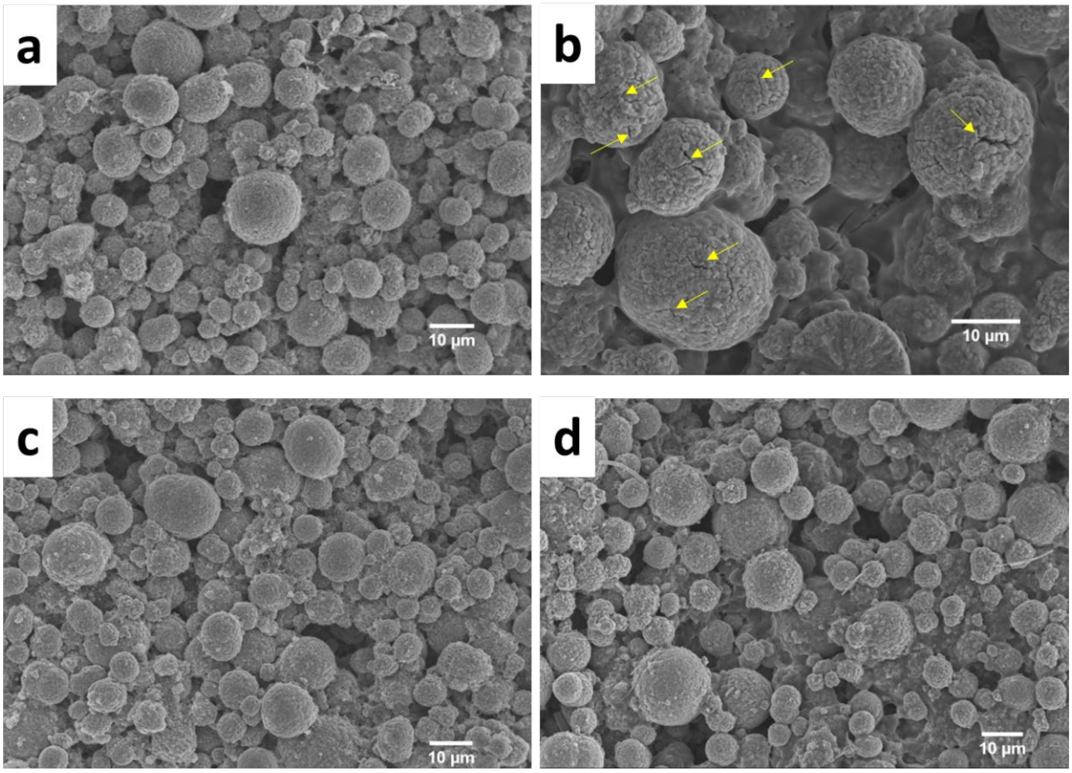

Figure S2 Scanning electron microscope (SEM) images of NMC particles in their as-coated/as-cycled states before particle dispersion treatment. (a) Pristine condition, (b) After 100 cycles with baseline electrolyte, (c) After 100 cycles with 0.5 wt.% VC containing electrolyte, and (d) After 100 cycles with 2 wt.% VC containing electrolyte.

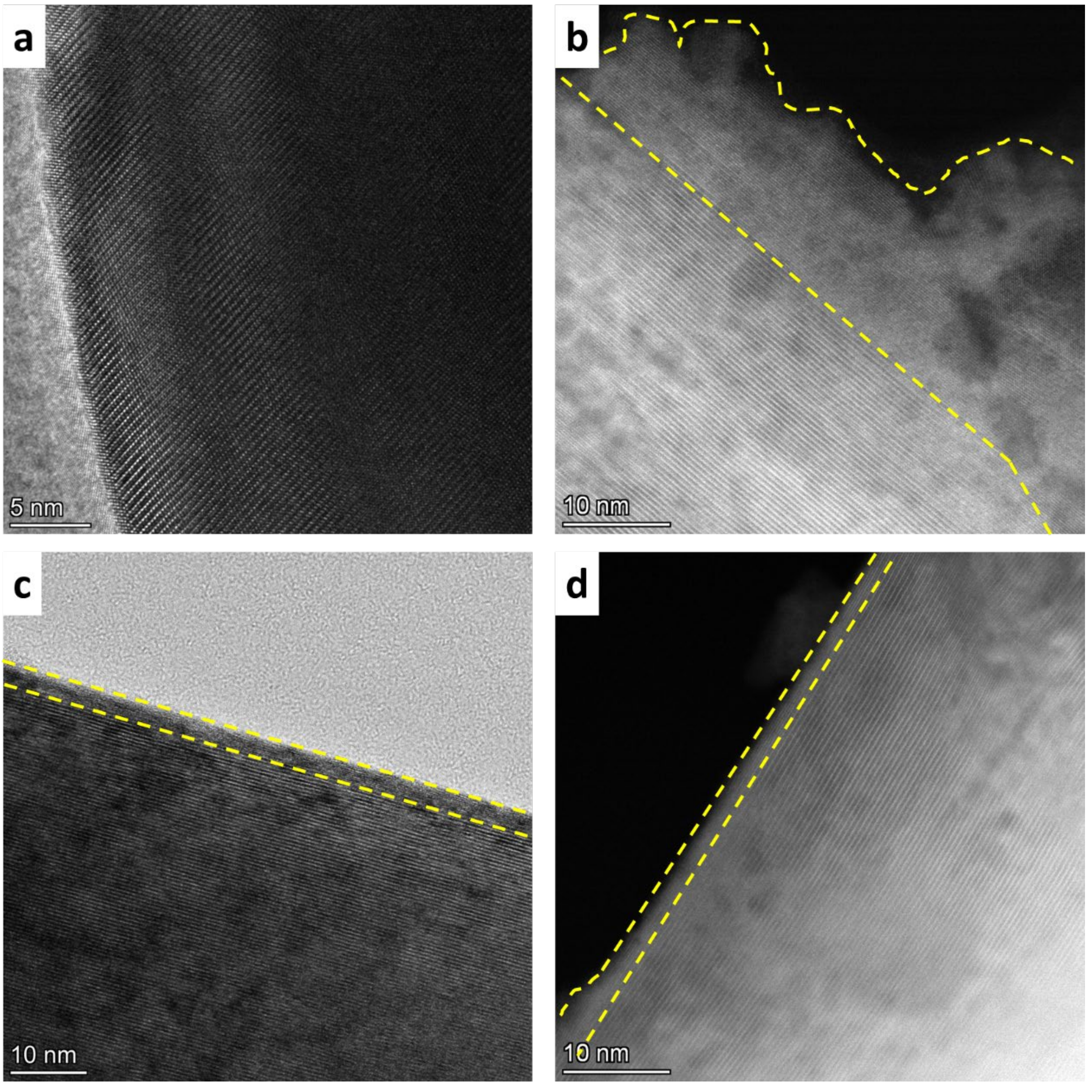

Figure S3 Transmission electron microscopy (TEM) and high-angle annular dark-field scanning transmission electron microscopy (HAADF-STEM) images of NMC particles at a lower magnification in (a) its pristine state, (b) after undergoing 100 cycles with the baseline electrolyte, (c) after 100 cycles with 0.5 wt.% VC containing electrolyte, and (d) after 100 cycles with 2 wt.% VC containing electrolyte. The rock-salt layer is indicated by yellow dashed lines. The rock-salt layer is indicated by yellow dashed lines.

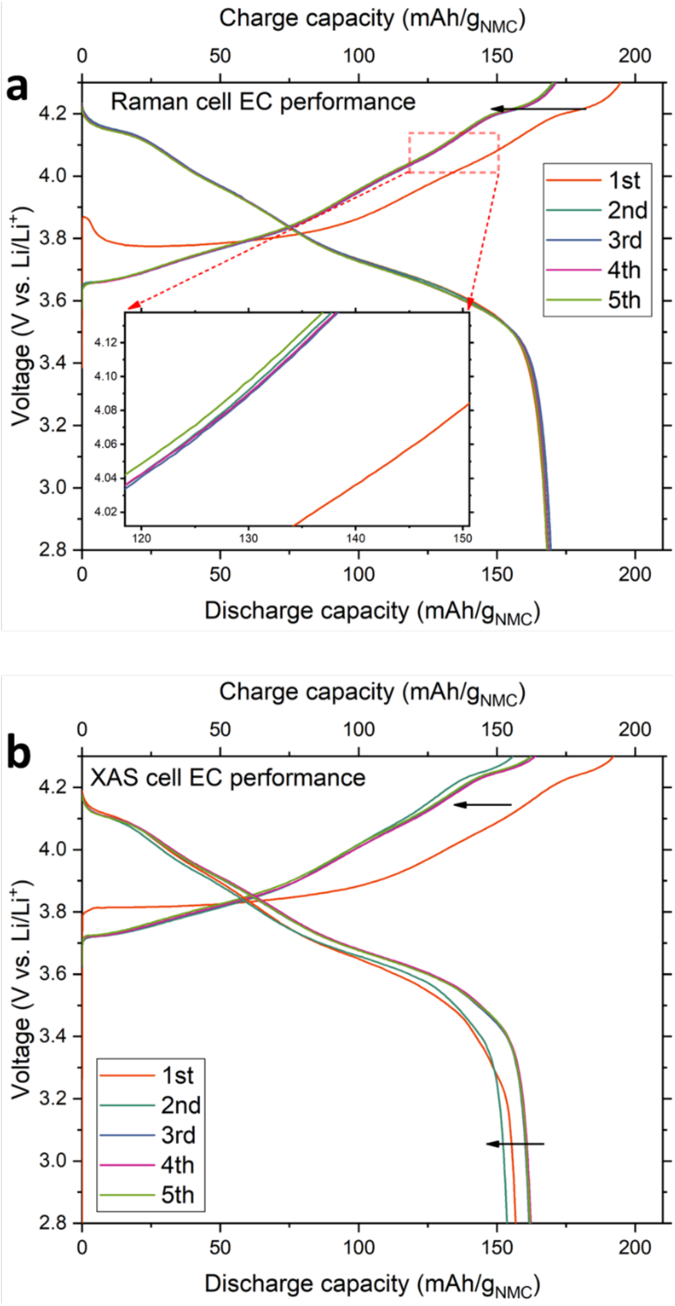

Figure S4 Chronopotentiometric cycling performance up to 5 cycles at C/10 between 2.8 and 4.3 V, as observed in (a) the in-situ/operando Raman cell and (b) the in-situ XAS cell. The inset in panel (a) exhibited the minor charge curves variation for the Raman cell. The cathode active materials were coated on separator (Celgard®) instead of Al foil for the purpose of spectroscopy experiments, therefore the performance showed relatively lower capacities compared to normal cells.

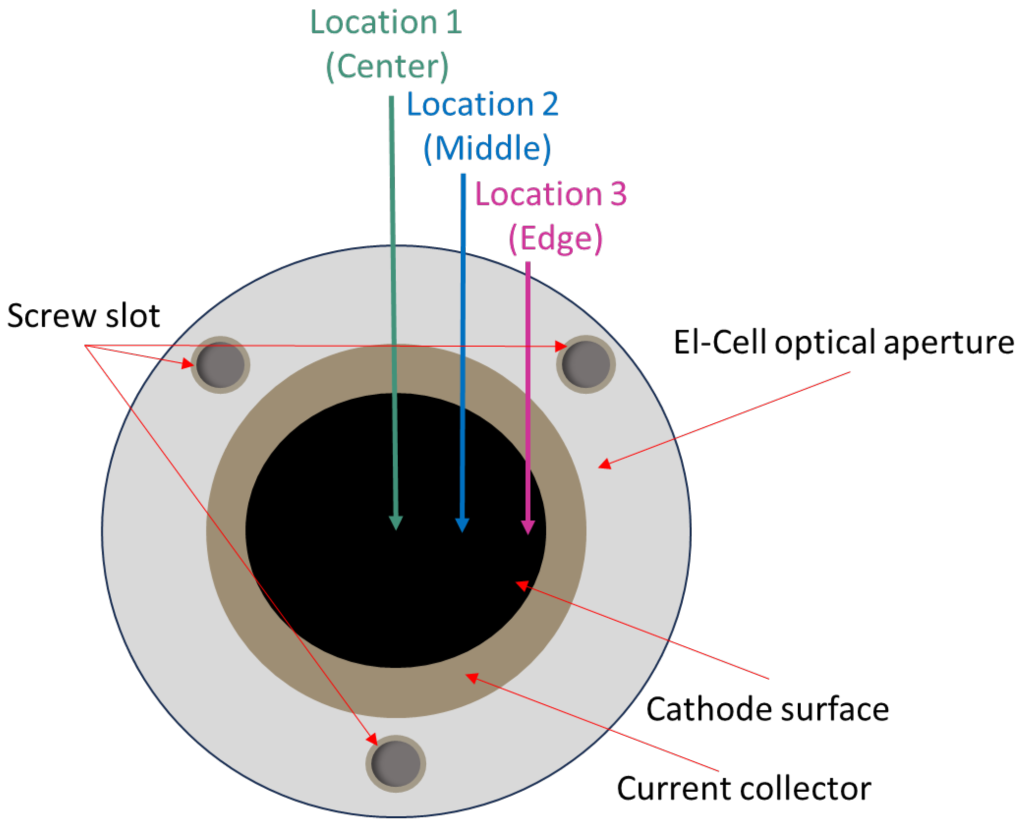

Figure S5 Schematic illustration of the top view of the Raman cell (El-Cell®) optical aperture. Various locations for Raman acquisition subsequent to formation cycles are marked using distinct colors: green denotes the center, blue signifies the middle, and pink designates the edge. Additionally, the other principal components are also labeled for reference.

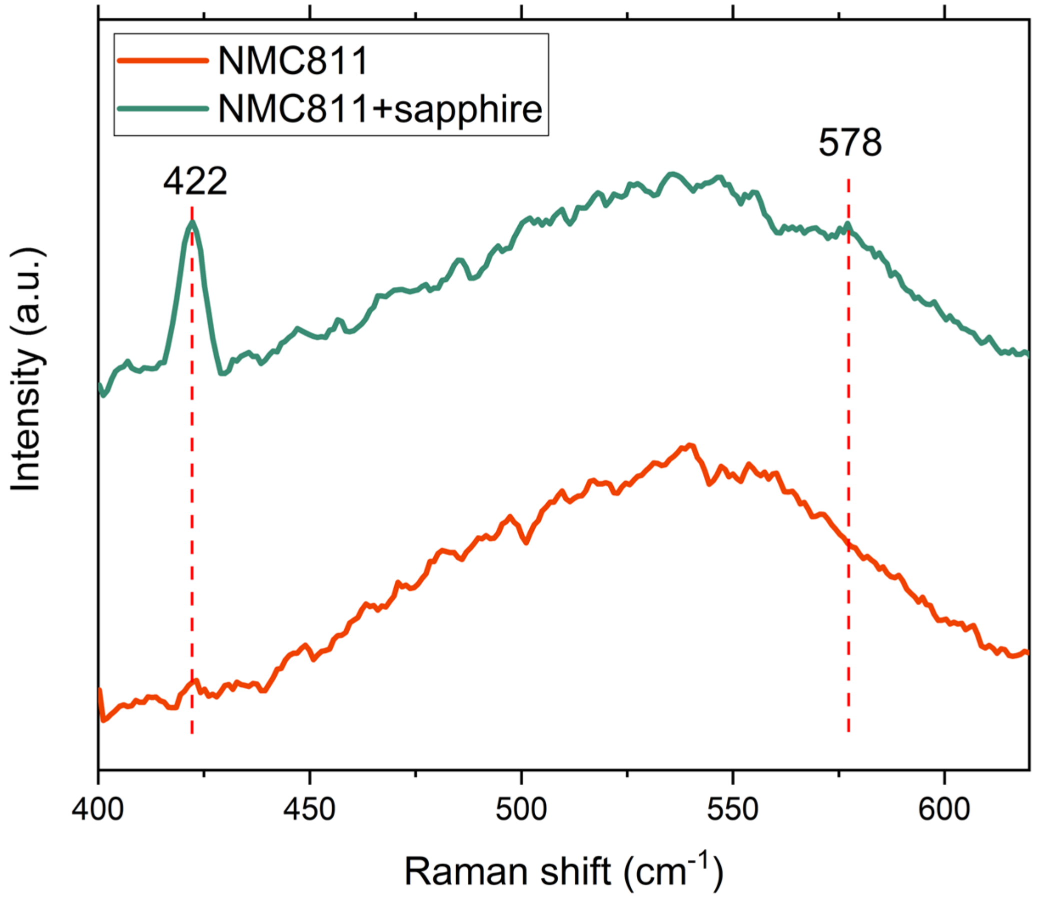

Figure S6 Comparison of ex-situ Raman spectra between pure NMC811 powders measured with a sapphire window (green) and without a sapphire window (red). The peaks at 422 and 578 cm^{-1} correspond to the stretching vibration (E_g mode) and bending mode (A_{1g} mode) of the Al-O, respectively, originating from the sapphire window.

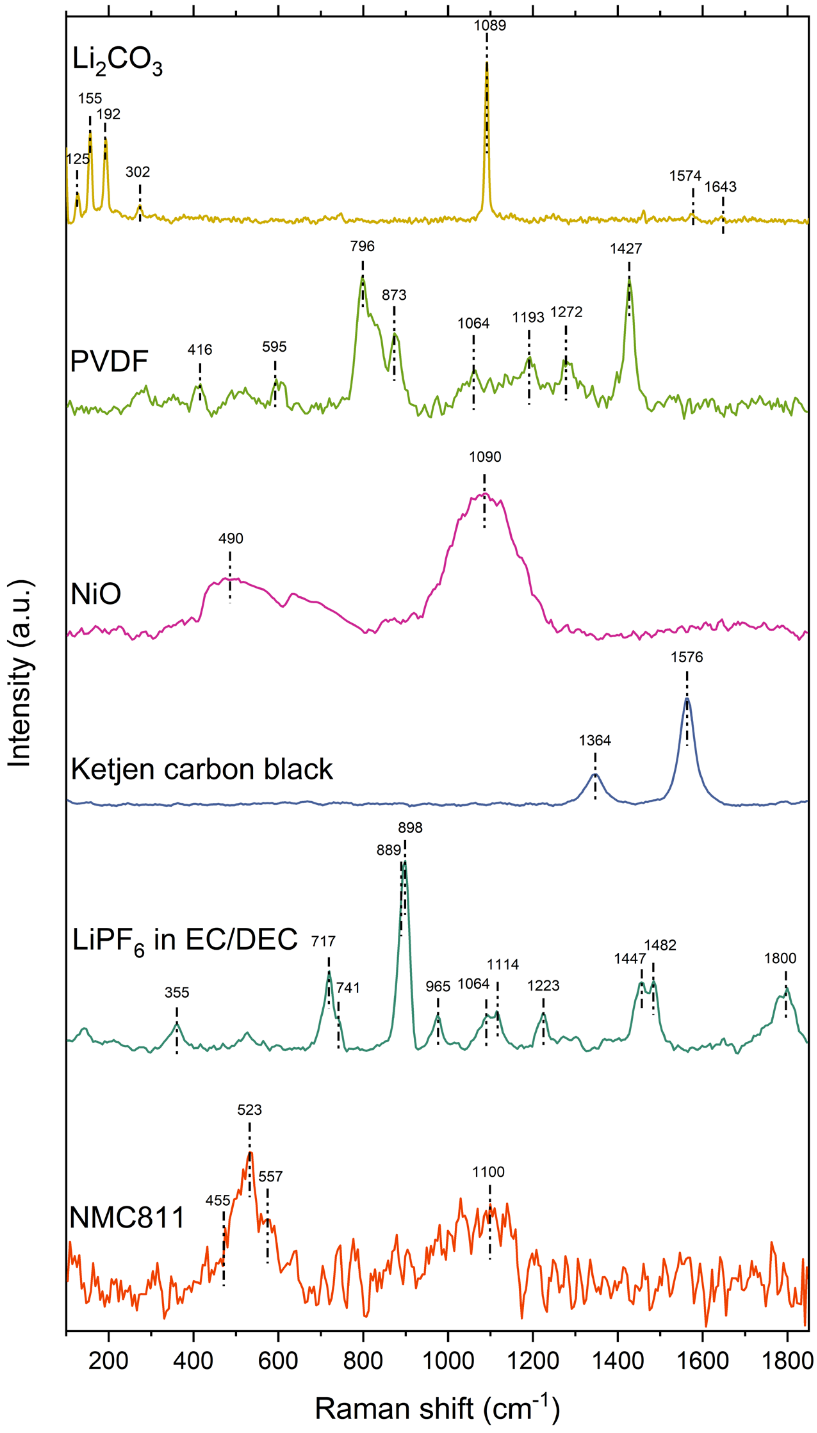

Figure S7 Ex-situ Raman spectra of various reference samples, including pure NMC811 powders, $LiPF_6$ solution in EC/DEC, pure Ketjen carbon black powders, pure NiO powders, pure PVDF powders, and pure Li_2CO_3 powers, as illustrated from bottom to top. The peak observed at 1090 cm^{-1} in the NiO reference sample

corresponds to the second-order longitudinal optical (2LO) mode[1]. Similarly, the broad peak at 1100 cm^{-1} corresponds to the transition metal second-order longitudinal optical (2LO) mode in NMC811 spectra: both of them arise due to multi-phonon excitation at resonance in the TM-O bonds.

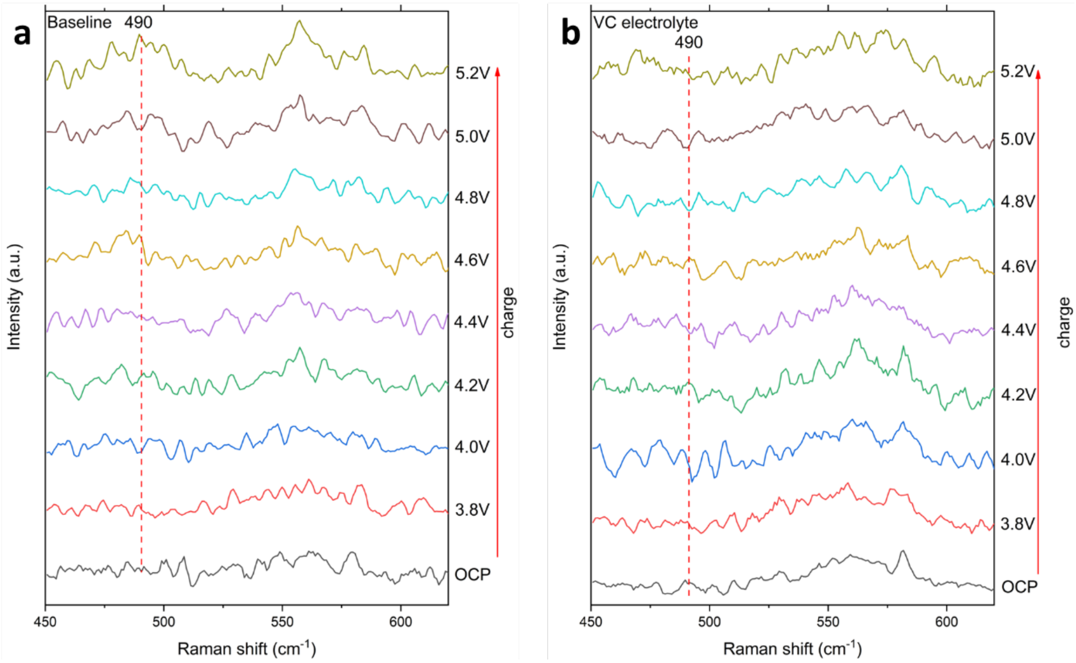

Figure S8 The normalized operando Raman spectra at each voltage stage from open circuit potential (OCP) conditions. Panel (a) shows the baseline electrolyte, while panel (b) displays the 2 wt.% VC additive electrolyte.

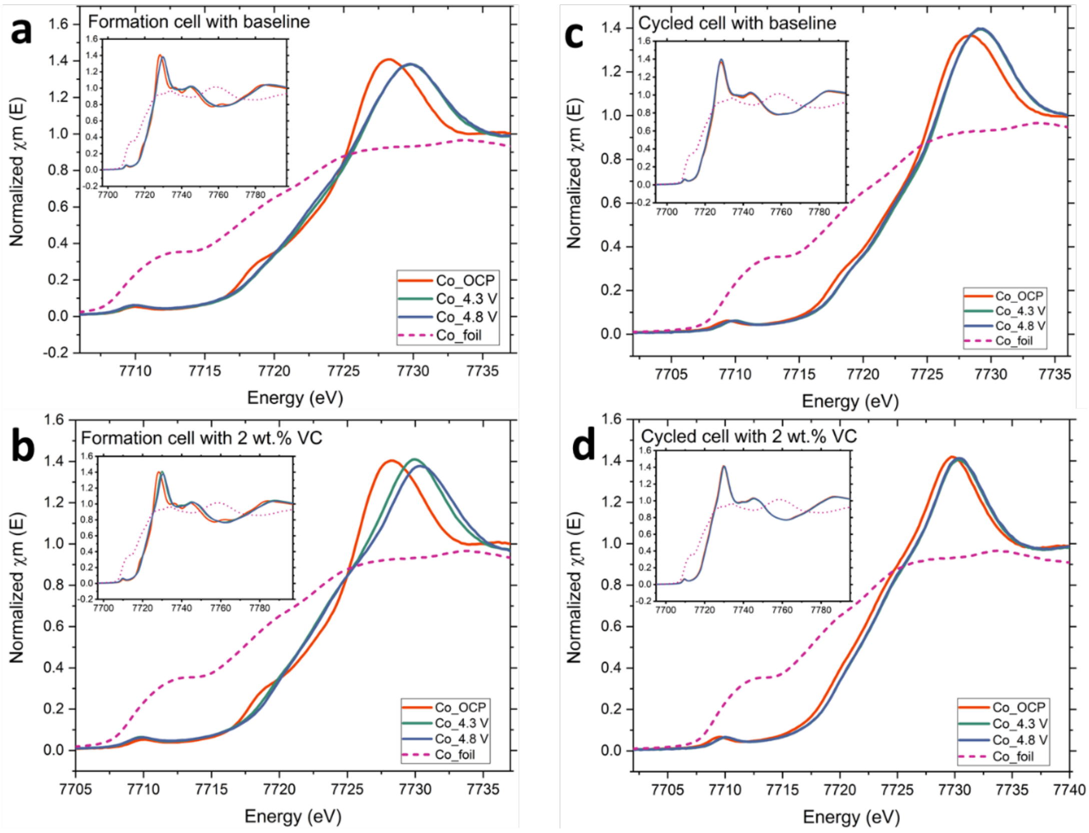

Figure S9 The normalized in-situ XANES spectra of Co K-edge during the first charge after formation cycles and extended cycles. Panel (a) represents the formation cell with the baseline electrolyte, panel (b) represents the formation cell with 2 wt.% VC, panel (c) represents the cycled cell with the baseline electrolyte, and panel (d) represents the cycled cell with 2 wt.% VC. The insets show the full range of the normalized XAS spectra for panels (a-d).

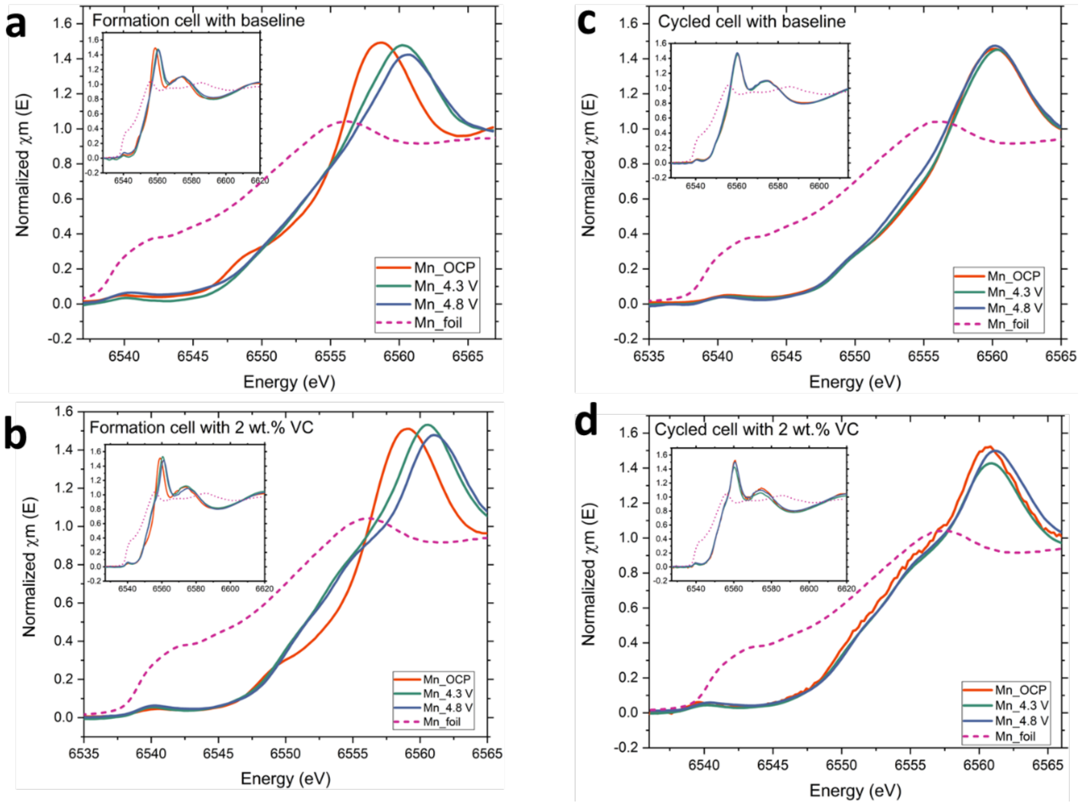

Figure S10 The normalized in-situ X-ray absorption near edge spectroscopy (XANES) spectra of Mn K-edge during the first charge after formation cycles and extended cycles. Panel (a) represents the formation cell with the baseline electrolyte, panel (b) represents the formation cell with 2 wt.% VC, panel (c) represents the cycled cell with the baseline electrolyte, and panel (d) represents the cycled cell with 2 wt.% VC. The insets show the full range of the normalized XAS spectra for panels (a-d).

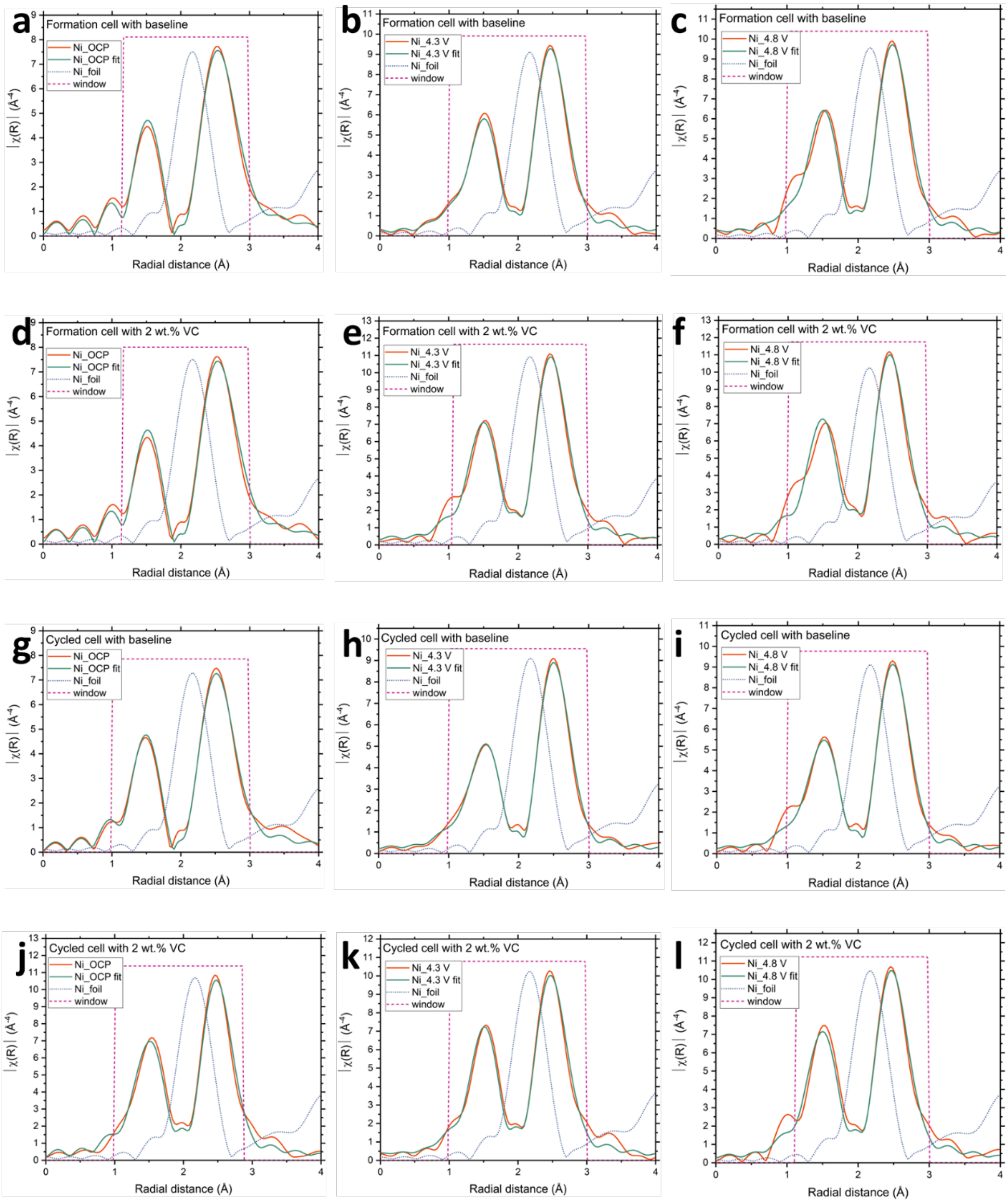

Figure S11 The Ni K-edge Fourier transform of extended X-ray absorption fine structure spectroscopy (FT-EXAFS) spectra including Ni^0 reference foil, corresponding fitting results, and fitting window for OCP, at 4.3 V, and at 4.8 V, respectively. Panel (a-c) displays formation cells with baseline electrolyte, and panel (d-f) displays formation cells with 2 wt.% VC electrolytes at 4th cycle (first cycle after formation cycles). While panel (g-i) represents cycled cells with baseline electrolyte,

and panel (j-k) represents cycled cells with 2 wt.% VC electrolytes at 21st cycle (first cycle after extended cycles). The specific fitting results are in Table S2.

Table S1 Summaries of Raman peaks from in-situ Raman spectra on cathode surface (Figure 3), where the VC related means peaks associated with VC polymerization or solvation with electrolyte.

**Substance**	**Raman shift (cm^{-1})**
Electrolyte	355, 717, 741, 889, 898, 966, 1114, 1447, 1482, 1800
Transition metals (TMs)	455, 523, 557
Sapphire	422
Binder/Electrolyte	1078, 1217
Carbon	1364, 1576
VC related	995, 1617
Li_2CO_3	1089

Table S2 Summaries of EXAFS fitting results of Ni at K-edge (Figure S11) at OCP, 4.3V and 4.8V during first charging cycle after formation cycles for (a-c) formation cells with baseline electrolyte, (d-f) formation cells with 2 wt.% VC electrolytes; after extended cycles for (g-i) cycled cells with baseline electrolyte, and (j-k) cycled cells with 2 wt.% VC electrolytes. *

Fig. index	Scattering	R(Å)	N	$\sigma^2 \times 10^{-3}$(Å^2)
**S11a**	Ni-O	1.954±0.009	2.063±0.121	3.7±1.2
	Ni-Ni	2.882±0.006	3.131±0.469	
**S11b**	Ni-O	1.890±0.007	2.654±0.121	3.4±0.8
	Ni-Ni	2.834±0.005	3.125±0.380	
**S11c**	Ni-O	1.885±0.008	2.702±0.148	3.2±1.2
	Ni-Ni	2.830±0.006	3.057±0.482	
**S11d**	Ni-O	1.954±0.010	2.056±0.134	3.9±1.3
	Ni-Ni	2.882±0.007	3.148±0.530	
**S11e**	Ni-O	1.880±0.007	2.701±0.134	2.3±0.9
	Ni-Ni	2.819±0.005	2.884±0.401	
**S11f**	Ni-O	1.876±0.010	2.683±0.184	2.0±1.3
	Ni-Ni	2.818±0.008	2.778±0.557	
**S11g**	Ni-O	1.916±0.007	2.304±0.148	4.7±0.8
	Ni-Ni	2.860±0.006	3.279±0.336	
**S11h**	Ni-O	1.907±0.006	2.451±0.097	4.1±0.7

	Ni-Ni	2.851±0.005	3.238±0.331	
**S11i**	Ni-O	1.901±0.007	2.612±0.113	4.1±0.8
	Ni-Ni	2.843±0.005	3.268±0.374	
**S11j**	Ni-O	1.880±0.008	2.681±0.154	2.4±1.2
	Ni-Ni	2.822±0.007	2.795±0.477	
**S11k**	Ni-O	1.880±0.009	2.631±0.155	2.5±1.2
	Ni-Ni	2.822±0.508	2.800±0.509	
**S11l**	Ni-O	1.878±0.008	2.733±0.164	4.7±0.8
	Ni-Ni	2.819±0.007	2.776±0.471	

*The FT-EXAFS fitting was done at Ni K-edge in R-space, $k^{1,2,3}$ weighting, with a window of $1.15<R<3$ Å. The fitting results of E_0 is in a range of 0.94±0.69 to 3.57±0.86 eV for different cells/conditions, S_0^2 was fixed at 0.784 for Ni K-edge obtained by fitting the corresponding reference foil.

Reference

(1) Lu, M.-L.; Lin, T.-Y.; Weng, T.-M.; Chen, Y.-F. Large enhancement of photocurrent gain based on the composite of a single n-type SnO_2 nanowire and p-type NiO nanoparticles. *Optics Express* **2011**, *19* (17), 16266-16272.
